# Mortality trends in type 1 diabetes: a multicountry analysis of six population-based cohorts

**DOI:** 10.1007/s00125-022-05659-9

**Published:** 2022-03-22

**Authors:** Paz L. D. Ruiz, Lei Chen, Jedidiah I. Morton, Agus Salim, Bendix Carstensen, Edward W. Gregg, Meda E. Pavkov, Manel Mata-Cases, Didac Mauricio, Gregory A. Nichols, Santa Pildava, Stephanie H. Read, Sarah H. Wild, Jonathan E. Shaw, Dianna J. Magliano

**Affiliations:** 1 Department of Chronic Diseases, Norwegian Institute of Public Health, Oslo, Norway; 2 Department of Endocrinology, Morbid Obesity and Preventive Medicine, Oslo University Hospital, Oslo, Norway; 3 Diabetes and Population Health, Baker Heart and Diabetes Institute, Melbourne, VIC Australia; 4 School of Public Health and Preventive Medicine, Monash University, Melbourne, VIC Australia; 5 Melbourne School of Population and Global Health, The University of Melbourne, Melbourne, VIC Australia; 6 Clinical Epidemiology, Steno Diabetes Center Copenhagen, Gentofte, Denmark; 7 Department of Epidemiology and Biostatistics, School of Public Health, Imperial College London, London, UK; 8 Division of Diabetes Translation, Centers for Diseases Control and Prevention, Atlanta, GA USA; 9 Centro de Investigación Biomédica en Red de Diabetes y Enfermedades Metabólicas Asociadas (CIBERDEM), Instituto de Salud Carlos III (ISCIII), Barcelona, Spain; 10 Institut Català de la Salut, Unitat de Suport a la Recerca Barcelona Ciutat, Institut Universitari d’Investigació en Atenció Primària Jordi Gol (IDIAP Jordi Gol), Barcelona, Spain; 11 Department of Endocrinology, Hospital de la Santa Creu i Sant Pau, Autonomous University of Barcelona, Barcelona, Spain; 12 Science Programs Department, Kaiser Permanente Center for Health Research, Portland, OR USA; 13 Research and Health Statistics Department, Centre for Disease Prevention and Control, Riga, Latvia; 14 Usher Institute, University of Edinburgh, Edinburgh, UK; 15 School of Life Sciences, La Trobe University, Melbourne, VIC Australia

**Keywords:** Consortium, Mortality, Non-communicable disease, Population health, Trends, Type 1 diabetes

## Abstract

**Aims/hypothesis:**

Mortality has declined in people with type 1 diabetes in recent decades. We examined how the pattern of decline differs by country, age and sex, and how mortality trends in type 1 diabetes relate to trends in general population mortality.

**Methods:**

We assembled aggregate data on all-cause mortality during the period 2000–2016 in people with type 1 diabetes aged 0–79 years from Australia, Denmark, Latvia, Scotland, Spain (Catalonia) and the USA (Kaiser Permanente Northwest). Data were obtained from administrative sources, health insurance records and registries. All-cause mortality rates in people with type 1 diabetes, and standardised mortality ratios (SMRs) comparing type 1 diabetes with the non-diabetic population, were modelled using Poisson regression, with age and calendar time as quantitative variables, describing the effects using restricted cubic splines with six knots for age and calendar time. Mortality rates were standardised to the age distribution of the aggregate population with type 1 diabetes.

**Results:**

All six data sources showed a decline in age- and sex-standardised all-cause mortality rates in people with type 1 diabetes from 2000 to 2016 (or a subset thereof), with annual changes in mortality rates ranging from −2.1% (95% CI −2.8%, −1.3%) to −5.8% (95% CI −6.5%, −5.1%). All-cause mortality was higher for male individuals and for older individuals, but the rate of decline in mortality was generally unaffected by sex or age. SMR was higher in female individuals than male individuals, and appeared to peak at ages 40–70 years. SMR declined over time in Denmark, Scotland and Spain, while remaining stable in the other three data sources.

**Conclusions/interpretation:**

All-cause mortality in people with type 1 diabetes has declined in recent years in most included populations, but improvements in mortality relative to the non-diabetic population are less consistent.

**Graphical abstract:**

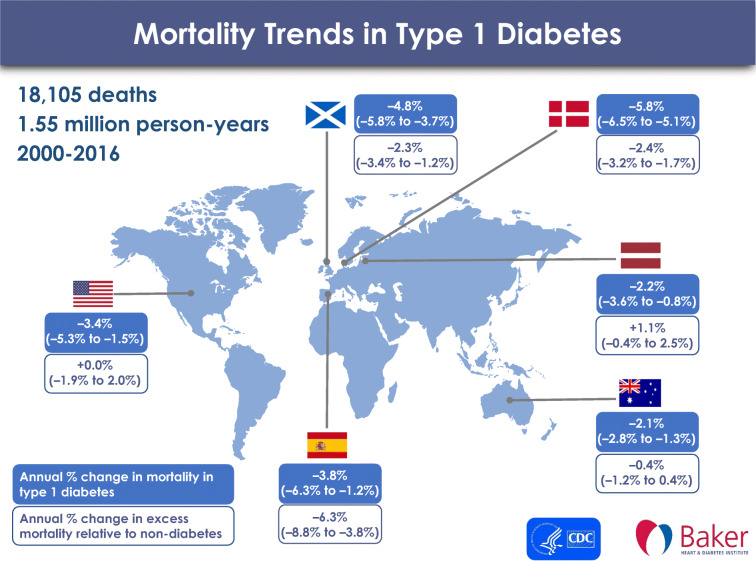

**Electronic supplementary material:**

The online version of this article (10.1007/s00125-022-05659-9) contains peer-reviewed but unedited supplementary material, which is available to authorised users.

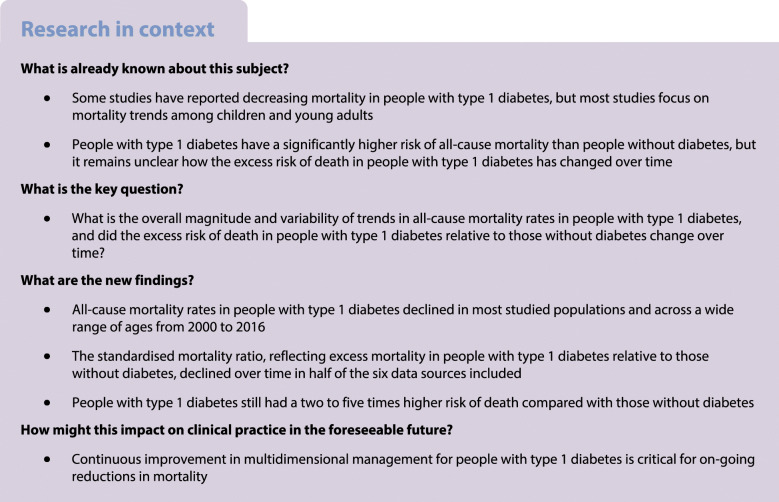



## Introduction

The overall age-standardised mortality in general populations, primarily from cardiovascular disease [1, 2], has been decreasing since the 1970s. In line with this, mortality in type 1 diabetes has also declined, primarily because of better diabetes care and prevention of complications [3–9]. However, most of the studies on mortality trends in type 1 diabetes focus on children and young adults [6, 9, 10], and there are few studies assessing mortality trends in type 1 diabetes across a broader age range [3–5, 7, 8]. Deaths in younger individuals with type 1 diabetes are rare, and are predominantly caused by acute complications. Secular mortality trends in middle-aged and older adults, for whom chronic complications dominate the causes of death [11, 12], and among whom the vast majority of deaths in type 1 diabetes occur, may differ markedly from those in younger adults. One earlier study reported the trends in cumulative mortality by calendar period of diagnosis in people with early and late onset of type 1 diabetes [13], but studies of age-specific trends in all-cause mortality in type 1 diabetes are scarce [4].

People with type 1 diabetes have a significantly higher risk of all-cause mortality than do people without diabetes [3, 8–10, 14–16]. However, it is not clear how this excess risk of all-cause death in people with type 1 diabetes has changed over time, and whether this has varied by sex, age or country.

Large observational studies with long follow-up times are needed to monitor these trends and to understand whether improvements in treatment have not only reduced all-cause mortality rates but have also narrowed the mortality gap between those with type 1 diabetes and the general population. Elucidating these mortality trends can help identify sub-populations in need of targeted intervention. Therefore, the aim of this study was to assess the trends in the absolute all-cause mortality rates among people with type 1 diabetes and trends in excess mortality in type 1 diabetes relative to those without diabetes in six countries, and to investigate whether the changes in both absolute and relative mortality rates over time varied by country, age and sex.

## Methods

### Data sources

This study used aggregate data on all-cause mortality in people with type 1 diabetes from an international diabetes consortium database, which has assembled longitudinal data on diabetes incidence and all-cause mortality from 24 predominantly administrative data sources in 21 countries or sub-national regions [17]. Of these datasets, seven included mortality data specifically for type 1 diabetes. After excluding data from Israel, which only had data for people with type 1 diabetes aged <50 years, we included data on people with type 1 diabetes aged 0–79 years from Australia, Denmark, Latvia, Scotland, Spain (Catalonia) and the USA (Kaiser Permanente Northwest [KPNW]) in the current analysis. Each data source provided aggregate data for each calendar year on population size, counts of prevalent and incident type 1 diabetes, death counts and person-years in people with type 1 diabetes and in people without diabetes, by sex and 5-year age-group (<20, 20–24, 25–29, …, 70–74, 75–79 years) from 2000 to 2016 (or a subset of this time period) (Table 1). This study was approved by the Human Ethics Committee of Alfred Health, Melbourne, Australia.

**Table 1:** Characteristics of the data sources

Country/region	Origin of data	Type of data	Years analysed for mortality	Person-years in people with type 1 diabetes (1000s)	Number of deaths in people with type 1 diabetes	Diabetes definition
Australia	National Diabetes Services Scheme	Registry	2004–2015	743	5727	Clinical diagnosis
Denmark	National Patient Register, prescription database, health insurance database, diabetes quality database, eye screening database	Registry	2005–2016	320	5898	Algorithm
Latvia	Latvian Diabetes Registry	Registry	2003–2016	54	1238	Clinical diagnosis (ICD-10)
Scotland	Scottish Care Information – Diabetes database	Registry	2006–2015	286	3819	Clinical diagnosis (Read codes)
Spain (Catalonia)	Information System for the Development of Research in Primary Care	Administrative	2009–2016	116	1031	Clinical diagnosis (ICD-10)
USA (KPNW)	KPNW (integrated managed care consortium)	Health insurance	2000–2016	27	392	Algorithm

For Read codes see https://digital.nhs.uk/article/1104/Read-Codes

### Assessment of diabetes status

Diabetes status was determined on the basis of clinical diagnosis by healthcare professionals or based on ICD-10 codes (http://apps.who.int/classifications/icd10/browse/2016/en) in the data from Australia, Latvia, Scotland and Spain (Catalonia), and using algorithms incorporating clinical diagnosis, linkage to medication or reimbursement registries and measurement of blood glucose or HbA_1c_ in Denmark and the USA (KPNW) data sources (Table 1 and electronic supplementary material [ESM] Table 1). Further, people were classified as having type 1 diabetes based on the ICD codes recorded in the database (Latvia, Spain [Catalonia] and the USA [KPNW] [18]) and criteria based on age at diagnosis or prescription history in addition to a diagnostic code of type 1 diabetes (Australia [19], Denmark [8] and Scotland [9]) (ESM Table 1).

### Outcome

Death in people with diabetes was determined by linkage to the death registries within each respective country or region. Sex- and age-specific numbers of deaths from any cause for the general population were obtained from the General Records of Incidence of Mortality (Australia), Statistics Denmark’s databank (Denmark), the National Records of Scotland’s mortality database (Scotland) and the Human Mortality Database (Latvia). For Spain (Catalonia) and the USA (KPNW), death in people without diabetes was determined by linkage to death registries.

### Quality of the included data sources

We assessed the quality of the included data and the risk of bias using a modified Newcastle−Ottawa scale [20] (details in ESM Methods). This modified scale included items that assess representativeness of the study population, the method of assessing diabetes status, whether gestational diabetes could be excluded, sample size at each time point, the method of assessing outcomes, and the number of data points (years) reported.

### Statistical analysis

We modelled mortality rates using age and calendar time as quantitative variables, scored as the midpoint of each age group (5 years) and calendar time interval (1 year). We used Poisson likelihood for multiplicative models, with death as the outcome and log person-years as offset. For the data source from Latvia, which did not have data on person-years in people with type 1 diabetes, we computed person-years in each year as the number of people with prevalent type 1 diabetes at the beginning of each year, plus half the number of people with incident type 1 diabetes in that year, minus half the number of deaths occurring in people with type 1 diabetes in that year. This computation assumes that new diabetes cases and deaths are uniformly distributed over each year. The numbers of deaths and person-years in people without diabetes reflect the population without any form of diabetes (Australia), or the population without type 1 or type 2 diabetes (other five data sources). We fitted age–period–cohort models [21] using cubic splines for the effects. Knots for the splines were placed at evenly spaced quantiles of the marginal distribution of the event times for each of the three variables in the model (age, period [calendar time] and cohort [period minus age]). Specifically, there were six knots for age, one knot per 4 years of period and four knots for cohort. For each data source and sex, we plotted the estimated mortality rates by age for a select set of dates 4 years apart, spanning the observation period, as well as mortality rates by period for five selected ages (40, 50, 60, 70 and 80 years). The estimated rates from the age–period–cohort models were used to calculate the age-standardised mortality rates using direct standardisation (using a type 1 diabetes population assembled from pooling the six data sources). We also fitted a set of age–period models with smooth age effects but a linear effect of calendar time for each data source, providing an overall summary of the annual changes in mortality rates for total people with type 1 diabetes and for male and female individuals separately. 95% CIs were computed as Wald CIs (back-transformed from log rates ±1.96 SE) [22]. We classified the mortality trend as ‘increasing’ if the point estimate of annual change was positive and the 95% CI excluded zero. Conversely, a trend was defined as ‘decreasing’ if the annual change was negative and its 95% CI excluded zero. When the 95% CI of the annual change included zero, we classified the trend as ‘unchanged’ (i.e. the change was not statistically significant).

Standardised mortality ratios (SMRs) were calculated by modelling the mortality rates in the entire population stratified by diabetes status, i.e. people with type 1 diabetes and people without diabetes [23]. An SMR of 1 indicates an equivalent mortality risk to the age- and sex-matched population without diabetes. A decline in SMR by calendar time implies that mortality rates among people with diabetes declined faster than among people without. The SMR was modelled in a similar way to the mortality rates, using Poisson likelihood for multiplicative models with observed number of deaths as outcomes and the log of expected number of deaths as the offset. As for mortality rates, we fitted models with a linear effect of calendar time for each data source, providing an overall summary of the annual changes in SMRs separately for each data source.

Stata software version 15.1 (Stata Corporation, College Station, TX, USA) was used for data management, and R software version 3.6.3 (R Foundation for Statistical Computing, Vienna, Austria) was used for statistical analyses and graphics.

## Results

Table 1 shows details for the six included data sources. All were from high-income countries: four European populations (Denmark, Latvia, Scotland and Spain), Australia and the USA. Four studies included national data, one study included regional data from Spain (Catalonia), and another study included data from a US regional health insurance database (KPNW) (Table 1). Quality scores for the data sources ranged from 5 to 9, with a median of 7 (IQR 6–8) (ESM Table 2).

There were 18,105 deaths (11,355 deaths in male individuals and 6750 in female individuals) during 1.55 million person-years of follow-up in 179,514 individuals with type 1 diabetes aged 0–79 years (Table 1, ESM Table 3). Overall, the ratio of male to female individuals with type 1 diabetes was 1.28 in the six included data sources, and the crude all-cause mortality rate was 11.7 (95% CI 11.5, 11.9) per 1000 person-years for all individuals with type 1 diabetes. The crude mortality rate was 13.2 (95% CI 12.9, 13.4) per 1000 person-years in male individuals, and 9.9 (95% CI 9.6, 10.1) per 1000 person-years in female individuals (ESM Fig. 1).

### Trends in all-cause mortality rates among people with type 1 diabetes

**Fig. 1:**
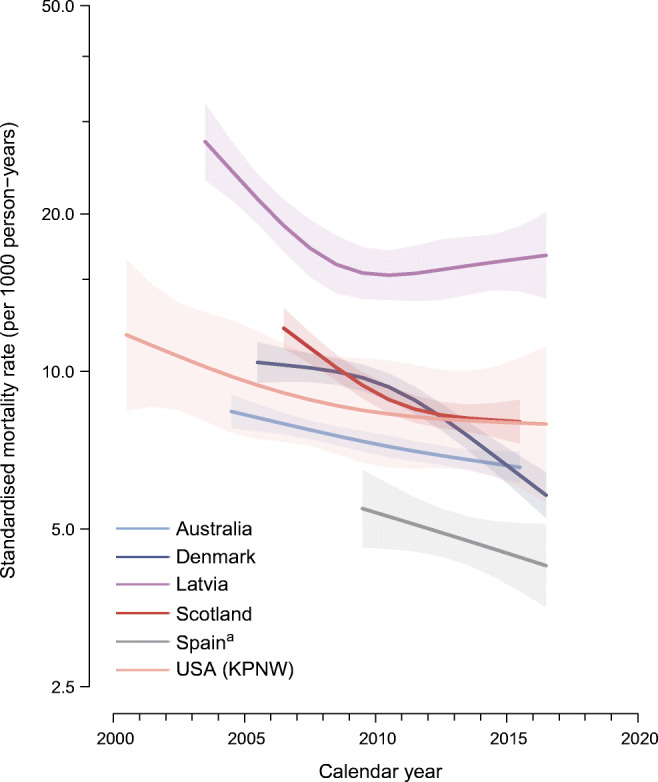
Age- and sex-standardised all-cause mortality rates in people with type 1 diabetes by calendar year. Standardisation is based on annual age-specific mortality rates from age–period–cohort models fitted separately for each data source and sex. The standard population was derived from the pooled study population with type 1 diabetes within the six data sources, with equal weights for male and female individuals. Shaded areas represent 95% CIs around mortality trends. The *y*-axis is plotted on a natural logarithmic scale. ^a^Data are from Catalonia, Spain

**Fig. 2:**
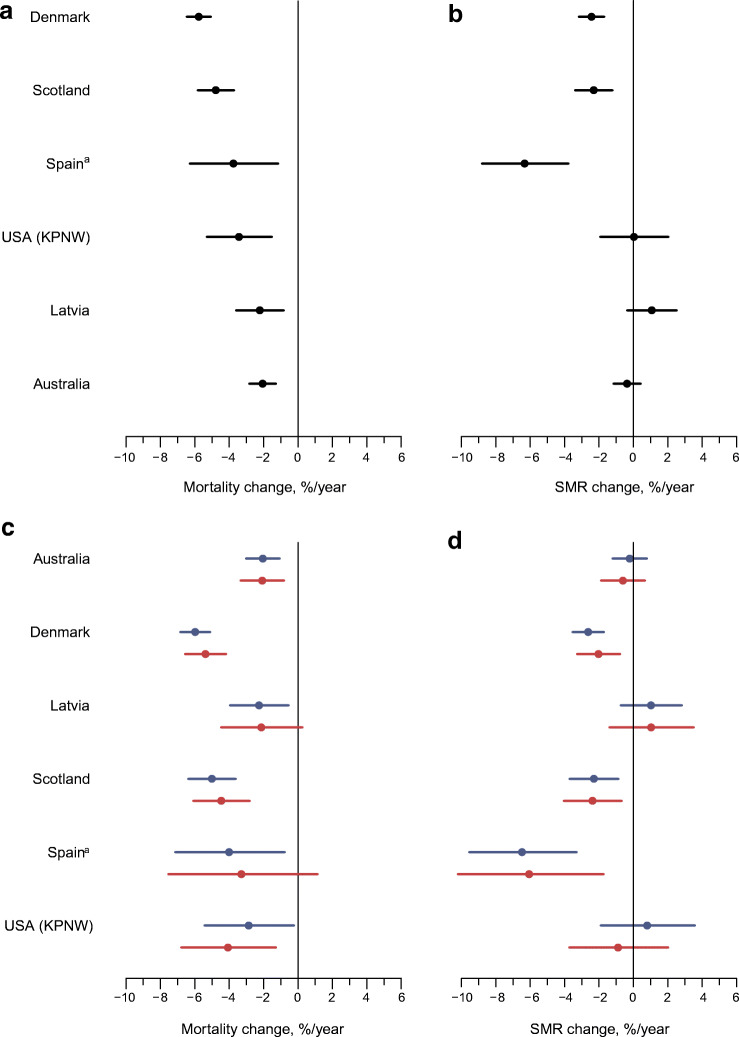
Annual estimated change in all-cause mortality rates in type 1 diabetes (**a**, **c**) and annual estimated change in SMR in type 1 diabetes relative to those without diabetes (**b**, **d**), in all individuals (**a**, **b**) and in male and female individuals separately (**c**, **d**). Data in (**a**, **b**) are ordered according to the magnitude of annual change in all-cause mortality rates in people with type 1 diabetes. Blue lines, male; red lines, female. Error bars indicate 95% CIs. ^a^Data are from Catalonia, Spain. SMR, standardised mortality ratio

The age- and sex-standardised all-cause mortality rate for each calendar year was highest in Latvia and lowest in Spain (Fig. 1, ESM Table 4). All six data sources showed a decline in the age- and sex-standardised all-cause mortality rates in people with type 1 diabetes over the whole study period, with annual estimated changes in mortality rates ranging from −2.1% (95% CI −2.8%, −1.3%) in Australia to −5.8% (95% CI −6.5%, −5.1%) in Denmark (Figs. 1 and 2a, ESM Table 5). In Latvia, the mortality remained relatively stable from approximately 2009 to 2016 (Fig. 1, ESM Table 4). Sex-specific mortality trends analysis showed that, over the whole study period, mortality declined in all male populations and in four female populations (Fig. 2c, ESM Fig. 2, ESM Table 5). Among Latvian female individuals, mortality fell for the first few years, and then rose until the end of the reporting period (2016), with an annual estimated change in mortality rates of −2.1% (95% CI −4.5%, 0.3%); among Spanish female individuals, the annual change in mortality rates was −3.3% (95% CI −7.5%, 1.1%).

### Trends in all-cause mortality rates in people with type 1 diabetes by age

ESM Figs. 3–8 present the age-specific and calendar time-specific all-cause mortality rates by sex for each data source. In general, all-cause mortality was higher for male individuals and for older individuals (ESM Figs. 2–8), but the rate of decline in mortality was generally unaffected by sex or age. An age by time interaction of mortality rates (estimated annual change in mortality by age) in each data source is shown in ESM Fig. 9. All-cause mortality rates decreased over time across ages in all data sources, except Latvia. Reductions in mortality rates over time appeared to be greater in people at older ages than younger ages for Australia and Denmark, and were similar across ages for the remainder.

### Trends in SMRs between people with type 1 diabetes and those without diabetes

Trends in SMRs in people with type 1 diabetes relative to people without diabetes are presented in Fig. 2b and ESM Table 5. The annual estimated change in SMR between type 1 diabetes and non-diabetes ranged from −6.3% (95% CI −8.8%, −3.8%) in Spain to 1.1% (95% CI −0.4%, 2.5%) in Latvia. Denmark, Scotland and Spain showed declines in SMRs over the whole study period, indicating larger declines in all-cause mortality rates among people with type 1 diabetes compared with those without diabetes. SMRs were stable in the other three data sources. Annual estimated changes in SMRs by sex were similar to those changes for male and female individuals combined (Fig. 2b,d, ESM Fig. 10 and ESM Table 5).

### Trends in SMRs in people with type 1 diabetes relative to those without diabetes by age and sex

**Fig. 3:**
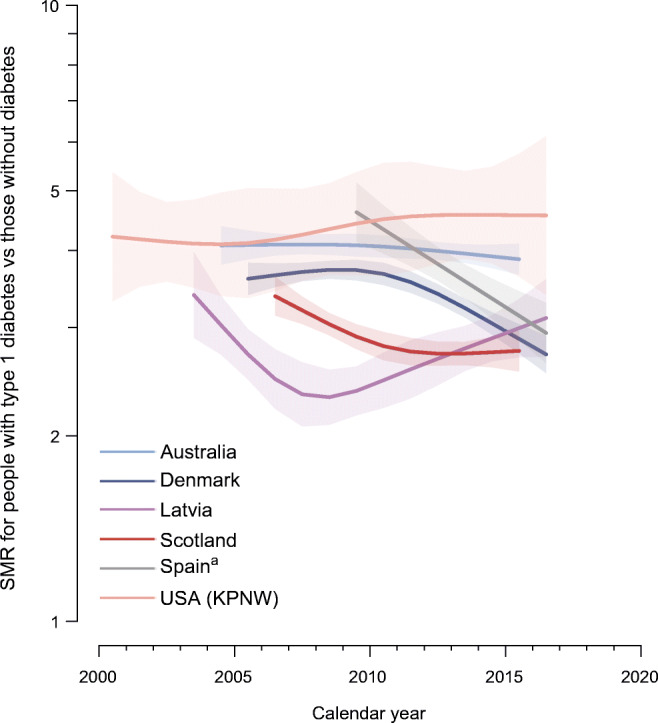
SMR in people with type 1 diabetes compared with those without diabetes by calendar year. Smoothing is based on a model with SMR constant over age and sex. Shaded areas represent 95% CIs. The *y*-axis is plotted on a natural logarithmic scale. ^a^Data are from Catalonia, Spain. SMR, standardised mortality ratio

ESM Figs. 11–16 present the age-specific and calendar time-specific SMR trends by sex for each data source. All-cause mortality rates were two to five times higher in people with type 1 diabetes than those without diabetes (Fig. 3), and were significantly elevated across all ages (ESM Figs. 11–16). For each data source, the SMR was higher in female individuals than male individuals at all ages for most calendar years, except in the USA (KPNW), which showed higher SMRs in older male individuals than older female individuals. In Denmark, SMR fell progressively with increasing age, but in other data sources, SMR rose to a peak at ages between 40 and 70 years old, and then fell. Denmark, Scotland and Spain showed a decrease in SMRs over the study period at all ages in both male and female individuals. Australia showed a decline in SMR among older individuals at ages 70 and 80 years but stable SMRs in younger individuals for both sexes. In Latvian female individuals, SMR initially fell, but then rose after about 2009. SMR was relatively stable across five selected ages in Latvian male individuals and in both sexes for the USA (KPNW).

## Discussion

Using contemporary aggregate data on all-cause mortality in people with type 1 diabetes from six data sources in high-income countries, we obtained four key findings. First, all data sources showed a decline in the age- and sex-standardised all-cause mortality rates in people with type 1 diabetes aged 0–79 years from 2000 to 2016 (or a subset thereof), with an annual estimated change in mortality rates ranging from −2.1% (95% CI −2.8%, −1.3%) to −5.8% (95% CI −6.5%, −5.1%). Furthermore, annual mortality rates declined in most country- and sex-specific populations, although this decline was not statistically significant in the Latvian and Spanish female populations. Second, mortality declined at a wide range of ages for most data sources. Third, the SMR, reflecting excess mortality, fell in half of the six included data sources. Fourth, despite reductions in absolute all-cause mortality rates, and, in some countries, in the SMR, people with type 1 diabetes still had a two to five times higher risk of death compared with those without diabetes.

Our observation of the decline in the age-standardised mortality rates in most populations with type 1 diabetes is consistent with previous studies from these countries for other time periods, with mortality declines among male and female populations with type 1 diabetes being reported in Australia (2000–2011) [4], Denmark (2005–2016) [8] and Scotland (from 2006–2010 to 2011–2015) [7]. Cardiovascular diseases are a major cause of deaths in individuals with type 1 diabetes [11, 12], although other important contributors to excess mortality include renal diseases, cancer and infectious diseases. Decreasing mortality in people with type 1 diabetes may be attributable to the advances in treatment and interventions for type 1 diabetes, as well as improvement in cardiovascular disease prevention with widespread use of statins and anti-hypertensive medications over the last two decades.

Despite reductions in all-cause mortality rates in people with type 1 diabetes in most populations studied, the improvement in the excess risk of all-cause death among people with type 1 diabetes relative to the non-diabetic population was less evident, with SMR decreasing in only three of the six data sources over the study period. Previous data from Australia showed that there was a reduction in excess all-cause mortality among both male and female individuals with type 1 diabetes aged 0–75 years between 1997 and 2010 [14]. A recent analysis of the Danish Diabetes Register reported that the SMR declined by approximately 2% per year for both sexes in the entire population with type 1 diabetes between 2005 and 2016 [8]. However, a cohort study from Sweden showed a decreased mortality rate among adults with type 1 diabetes from 1998 to 2014, but they did not find a similar decline in the excess mortality relative to the general population [5].

Similar to other studies [3], absolute mortality rates in people with type 1 diabetes increased with increasing age, while the excess mortality for type 1 diabetes relative to those without diabetes decreased with increasing age. As has been reported previously [9, 16], we found that the SMR associated with type 1 diabetes was higher among female individuals than male individuals across all ages for most calendar years. We also noted that mortality declined over the study period across most ages for both male and female individuals, while SMR declined at all ages examined in only three out of six data sources. Studies of age-specific trends in all-cause mortality rates or excess mortality in people with type 1 diabetes are scarce, and most were restricted to younger individuals with type 1 diabetes [6, 9, 10, 14]. Previous work from our group indicated that age-specific SMRs in people with type 1 diabetes in Australia did not significantly change between 1997–2003 and 2004–2010 [14]. A cohort study of individuals diagnosed with type 1 diabetes before the age of 15 years from Northern Ireland did not find a significant change in either all-cause mortality rates or corresponding SMR associated with type 1 diabetes from 1989 to 2012 [10]. Studies in Uzbekistan (1998–2014) [6] and Scotland (2004–2017) [9] identified mortality reductions in children <15 years and in people aged below 50 years, respectively. However, there was no improvement in excess mortality for individuals with type 1 diabetes under age 50 years in Scotland from 2004 to 2017 [9].

Despite reductions in absolute all-cause mortality rates, and, in some countries, in the SMR, type 1 diabetes still confers a higher excess risk of death compared with individuals without diabetes. Suboptimal glycaemic control and the presence of acute and chronic complications are key contributors to excess risk of death in type 1 diabetes [3, 9, 15]. Even among people with type 1 diabetes who have an HbA_1c_ below the target level of 53 mmol/mol (7.0%), the risk of all-cause mortality is still twice that of the general population [15]. Evidence shows that intensive insulin therapy is associated with a decreased all-cause mortality compared with conventional therapy, with a persistent benefit more than 30 years later [24, 25]. However, data from the USA T1D Exchange Clinic Network showed that there was an increase in mean HbA_1c_ from 62 mmol/mol (7.8%) to 68 mmol/mol (8.4%) between 2010–2012 and 2016–2018 [26]. More recent Scottish data showed that, despite an overall declining trend in HbA_1c_ level from 70 mmol/mol (8.6%) to 68 mmol/mol (8.4%) in people with type 1 diabetes (2012–2016), more than one-third of all those with type 1 diabetes still had poor glycaemic control with an HbA_1c_> 75 mmol/mol (>9%) in 2016 [27]. Poor glycaemic control in younger people with type 1 diabetes increases the risk of developing complications when they age [25], and increases the risk of death from any cause or from cardiovascular causes [15]. In addition to glycaemic management, data from the Swedish National Diabetes Register suggested a steep increase in the excess risk of all-cause death with decreasing number of cardiovascular risk factors (BP, LDL-cholesterol, smoking and albuminuria) meeting target levels among people with type 1 diabetes [28].

### Strengths and weaknesses

A key strength of this work is that we have assembled six large population-based cohorts, with a sizeable number of people with type 1 diabetes and a long study period to enable us to provide estimates of time trends in all-cause mortality rates in six countries, and by sex and age categories. To the best of our knowledge, this work is the only study of this kind, and provides the most recent data on mortality trends in people with type 1 diabetes across a broader age range. Four out of six sources were national diabetes registries, which cover the entire population with type 1 diabetes in those countries.

Several potential limitations of our study should be considered. First, misclassification of diabetes type cannot be ruled out. Algorithms based on clinical criteria, medication use and laboratory measurements are commonly used in administrative databases and diabetes registries to assign diabetes type. However, the absence of some laboratory data from the database, such as autoantibody or C-peptide levels, may lead to misclassification of diabetes type. The use of algorithms to classify type 1 diabetes based on age at diagnosis in some data sources may exclude those with older-onset type 1 diabetes [29], which means that the mortality rates in type 1 diabetes may be underestimated. However, within each data source, the same approach was used to classify diabetes type across time periods, therefore mortality trends are less likely to be affected by the potential misclassification of older-onset type 1 diabetes. Second, we did not have access to individual clinical data. Therefore, we could not determine whether mortality trends in people with type 1 diabetes were due to changes in glycaemic control, changes in other risk factors, or differences in the prevalence of acute or chronic diabetes-related complications over time. Moreover, improved survival over time will increase diabetes duration, an important risk factor for mortality, which may have attenuated improvements in age-specific mortality rates. We did not have access to data on age at diagnosis of type 1 diabetes or diabetes duration, and thus could not examine the relationship of mortality with duration of diabetes. As data were not available for risk factors in people with and without diabetes, we were not able to investigate possible explanations for the excess risk of mortality among people with type 1 diabetes. Third, the sample size was variable between the six data sources, with a relatively small number of people with type 1 diabetes in the data sources from Spain (Catalonia), the USA (KPNW) and Latvia. Fourth, the six data sources only represent high-income countries; therefore, our results may not be generalisable to middle- and low-income countries where patterns of mortality trends in people with type 1 diabetes remain uncertain. Fifth, we did not have data on ethnicity, socioeconomic status or population immigration. Finally, our data are limited in terms of the time period covered by some data sources.

### Conclusions

This is the first multicountry analysis of six large contemporary population-based studies, and shows that all-cause mortality in people with type 1 diabetes has declined in recent years in most studied populations. However, excess mortality relative to the population without diabetes remains high in people with type 1 diabetes. Considering the increasing incidence of type 1 diabetes observed in younger populations in recent years [8, 30, 31], it is critical to continuously improve the multidimensional management of type 1 diabetes, particularly among younger populations.

## Supplementary Information

ESM 1 (PDF 1.27 mb)

## Data Availability

Aggregate data may be available upon reasonable request to the corresponding author. There might be limitations on what the data can be used for, subject to approval from the data custodians.

## Abbreviations

KPNW: Kaiser Permanente Northwest
SMR: Standardised mortality ratio
