# Supplementary material for: Mortality trends in type 1 diabetes: a multicountry analysis of six population-based cohorts
Source: Diabetologia. 2022 Mar 22;65(6):964–72. doi: 10.1007/s00125-022-05659-9 (PMC9076725; doi:10.1007/s00125-022-05659-9)
Supplement: Supplementary file 1 — (PDF 1.27 mb) [file 125_2022_5659_MOESM1_ESM.pdf]

Electronic Supplemental Material (ESM)

This document provides supplementary material for the manuscript: Mortality trends in type 1 diabetes: a multicountry analysis of six population-based cohorts

Table of contents

ESM Methods ……..……………………………………………………………………3

ESM Table 1 Diabetes definition by data source 4

ESM Table 2 Quality assessment of the included data sources...6

ESM Table 3 Crude all-cause mortality rates by data source and diabetes status in male and female individuals aged 0-79 years, separately 7

ESM Table 4 All-cause mortality rates in people with type 1 diabetes aged 0-79 years for each calendar year..8

ESM Table 5 Annual estimated change in all-cause mortality rates in people with type 1 diabetes and annual estimated change in SMR..10

ESM Fig. 1 Crude all-cuase mortality rates in people with type 1 diabetes stratified by sex. 11

ESM Fig. 2 Age-standardised all-cause mortality rates in people with type 1 diabetes stratified by sex 12

ESM Fig. 3 Estimated mortality rates in people with type 1 diabetes by age and period in Australia. 13

ESM Fig. 4 Estimated mortality rates in people with type 1 diabetes by age and period in Denmark. 14

ESM Fig. 5 Estimated mortality rates in people with type 1 diabetes by age and period in Latvia. 15

ESM Fig. 6 Estimated mortality rates in people with type 1 diabetes by age and period in Scotland. 16

ESM Fig. 7 Estimated mortality rates in people with type 1 diabetes by age and period in Spain 17

ESM Fig. 8 Estimated mortality rates in people with type 1 diabetes by age and period in the USA (KPNW) 18

ESM Fig. 9 Age by time interaction of mortality rates as estimated annual change in mortality by age 19

ESM Fig. 10 SMR in people with type 1 diabetes compared with those without diabetes stratified by sex 20

ESM Fig. 11 Estimated SMR in people with type 1 diabetes compared with those without diabetes by age and period in Australia ……………………………………..………………21

ESM Fig. 12 Estimated SMR in people with type 1 diabetes compared with those without diabetes by age and period in Denmark..22

ESM Fig. 13 Estimated SMR in people with type 1 diabetes compared with those without diabetes by age and period in Latvia ……………………………………………………..23

ESM Fig. 14 Estimated SMR in people with type 1 diabetes compared with those without diabetes by age and period in Scotland..24

ESM Fig. 15 Estimated SMR in people with type 1 diabetes compared with those without diabetes by age and period in Spain...25

ESM Fig. 16 Estimated SMR in people with type 1 diabetes compared with those without diabetes by age and period in the USA (KPNW) 26

References..27

ESM Methods

Modified Newcastle-Ottawa Quality Assessment Scale for mortality trends in type 1 diabetes.

A study can be awarded a maximum of one or two points for each numbered item within each category.

Selection

1. Representativeness of the general population (sampling frame).

   a) National scheme with ≥80% coverage of national population (2 points)
   b) Random sample from national health insurance or national population-based survey with ≥80% response rate (1 point)
   c) Regional representative or national scheme with <80% coverage of national population (0 points)

2. Assessment of diabetes status.

   a) By blood glucose measurement (FPG, OGTT, HbA_{1c}) or by multiple approaches / administrative algorithm where 2 or more criteria used (2 points)
   b) Clinical diagnosis (e.g. ICD code or physician-diagnosed) (1 point)
   c) Anti-diabetic medication or self-report of physician-diagnosed diabetes (0 points)

3. Exclusion of gestational diabetes

   a) Yes (1 point)
   b) No (0 points)

4. Sample size at each time point.

   a) >5,000 (1 point)
   b) ≤5,000 (0 points)

Outcome

1. Assessment of outcome

   a) By record linkage (1 point) e.g. national death registry
   b) Other – regular follow-up (0 points)

Completeness of trend data

1. How many time points are provided?

   a) ≥10 (2 points)
   b) 6 – 9 (1 point)
   c) <6 (0 points)

Total score = 9

ESM Table 1 Diabetes definition by data source

Country/ region	Diagnostic method	Classification of diabetes type
Australia	Clinical diagnosis certified by a doctor, nurse or credentialed diabetes educator.	Persons were classified as having type 1 diabetes if they were registered as type 1 diabetes on the NDSS, had evidence of ongoing treatment with insulin (≥2 prescriptions on the PBS, except where time to census date/death was <2 years), and met one of the following additional criteria [1]:<br>1) time between diagnosis date and first insulin prescription was less than one year, or<br>2) registered before 45 years of age and showing evidence of insulin therapy at the time of registration (only applied to individuals with missing diagnosis date [31%]); or<br>3) registered before 2002 with an age at diagnosis <30 years (or age at registration <45 years if missing diagnosis date) and treatment with insulin evident from 2002; i.e. when PBS data became available (only applied to individuals with missing insulin initiation date in the NDSS database).<br>In addition, registrants who were originally classified as type 2 on the NDSS, but whose age at diagnosis was <30 years and time to insulin was <1 year, were reclassified as type 1 diabetes.
Denmark	Algorithm incorporating clinical diagnosis (ICD codes) from the hospitalisations or outpatient clinics, prescription of anti-diabetic medications, clinical and billing records.	Persons were classified as having type 1 diabetes in the diabetes register if any of the following criteria were met, and otherwise as type 2 diabetes:<br>1) Purchase of insulin before age 30, or<br>2) DADD: classified as having type 1 diabetes in >50% of the person's DADD records classify the person as type 1 diabetes, and similarly for type 2 diabetes, or<br>3) Not classified as either type 1 or type 2 diabetes in DADD, but >50% of the patient's records from National Patient Register classifies the person as having type 1 diabetes.<br>Finally, a person cannot be classified as having type 1 diabetes if there is no recorded date of insulin purchase [2].

Country/ region	Diagnostic method	Classification of diabetes type
Latvia	Clinical diagnosis using ICD-10 codes.	Persons were classified as having type 1 diabetes according to ICD-10 codes.
Scotland	Clinical diagnosis using the Read code system.	Persons were classified as having type 1 diabetes using clinician-assigned diabetes type for each individual, which was accepted unless contradictory prescription history or age-at-diagnosis data were available (e.g. a clinician-assigned diagnosis of type 1 diabetes without any subsequent insulin prescription would not be accepted) [3].
Spain (Catalonia)	Clinical diagnosis using ICD-10 codes.	Persons were classified as having type 1 diabetes according to ICD-10 codes.
USA (KPNW)	Algorithm incorporating hospitalisation with diabetes as primary discharge diagnosis (ICD codes), ≥2 outpatient visits (ICD codes), anti-diabetic medications or two abnormal blood results from an integrated healthcare delivery system.	Persons were classified as having type 1 diabetes if >50% of the person's records had a diagnosis code of type 1 diabetes within a 2-year period [4].

DADD, Danish Adult Diabetes Database; ICD-10, International Classification of Diseases, version 10; NDSS, National Diabetes Services Scheme; PBS, Pharmaceutical Benefits Scheme.

ESM Table 2 Quality assessment of the included data sources

Country/ region	Origin of data	Represen-tativeness of population	Assessment of diabetes status	Exclusion of gestational diabetes	Sample size at time points (no. of type 1 diabetes)	Assessment of outcome	Completeness (no. of data points)	Total Score
Range of allocated points		0-2	0-2	0-1	0-1	0-1	0-2	9
Australia	National Diabetes Services Scheme	2	1	1	1	1	2	8
Denmark	National Patient Register, prescription data base, health insurance data base, diabetes quality database, and eye screening data base	2	2	1	1	1	2	9
Latvia	Latvian Diabetes Registry	2	1	1	0	1	2	7
Scotland	Scottish Care Information-Diabetes database	2	1	0	1	1	2	7
Spain (Catalonia)	Information System for the Development of Research in Primary Care	0	1	1	1	1	1	5
USA (KPNW)	KPNW (integrated managed care consortium)	0	2	1	0	1	2	6

ESM Table 3 Crude all-cause mortality rates by data source and diabetes status in male and female individuals aged 0-79 years, separately

	With type 1 diabetes			Without diabetes		
	Number of deaths	Person-years (1000s)	Crude rate (per 1000 person-years)	Number of deaths	Person-years (1000s)	Crude rate (per 1000 person-years)
**Male**						
Australia	3587	408	8.8	404491	122722	3.3
Denmark	3851	182	21.1	151046	30783	4.9
Latvia	811	30	27.4	155529	13028	11.9
Scotland	2231	161	13.9	133778	23501	5.7
Spain[a]	666	66	10.0	68610	20380	3.4
USA (KPNW)	209	14	15.1	11010	3560	3.1
**Female**						
Australia	2140	335	6.4	260087	121584	2.1
Denmark	2047	138	14.9	112537	30812	3.7
Latvia	427	24	17.6	94686	14623	6.5
Scotland	1588	125	12.7	103631	24766	4.2
Spain[a]	365	50	7.3	37289	20481	1.8
USA (KPNW)	183	13	14.1	9376	3750	2.5

[a]Data are from Catalonia, Spain.

ESM Table 4 All-cause mortality rates in people with type 1 diabetes aged 0-79 years for each calendar year

Country/ region	Calendar year	Number of deaths	Crude rate (per 1000 person-years)	Age- and sex-standardised rate (per 1000 person-years, 95% CI)
Australia	2004	383	7.5	8.4 (7.8, 9.0)
	2005	366	7.0	8.2 (7.7, 8.7)
	2006	401	7.4	7.9 (7.6, 8.3)
	2007	417	7.4	7.7 (7.4, 8.1)
	2008	463	7.9	7.5 (7.2, 7.9)
	2009	466	7.7	7.4 (7.0, 7.7)
	2010	462	7.3	7.2 (6.9, 7.5)
	2011	530	8.1	7.0 (6.7, 7.4)
	2012	501	7.5	6.9 (6.6, 7.2)
	2013	549	7.9	6.8 (6.5, 7.1)
	2014	570	8.0	6.7 (6.3, 7.0)
	2015	619	8.4	6.6 (6.1, 7.0)
Denmark	2005	586	23.3	10.4 (9.5, 11.4)
	2006	576	22.7	10.3 (9.6, 11.1)
	2007	571	22.3	10.2 (9.5, 10.8)
	2008	562	21.7	10.0 (9.4, 10.6)
	2009	564	21.6	9.7 (9.2, 10.3)
	2010	551	20.8	9.3 (8.8, 9.9)
	2011	493	18.4	8.8 (8.3, 9.3)
	2012	440	16.3	8.2 (7.7, 8.7)
	2013	417	15.3	7.5 (7.1, 8.0)
	2014	377	13.6	6.9 (6.4, 7.4)
	2015	375	13.4	6.3 (5.8, 6.9)
	2016	386	13.6	5.8 (5.2, 6.4)
Latvia	2003	97	29.9	27.5 (23.2, 32.5)
	2004	89	26.7	24.2 (21.3, 27.5)
	2005	90	25.8	21.4 (19.1, 23.8)
	2006	96	26.5	19.0 (16.9, 21.3)
	2007	70	18.8	17.2 (15.2, 19.5)
	2008	83	21.6	16.0 (14.1, 18.2)
	2009	87	22.2	15.4 (13.8, 17.3)
	2010	77	19.6	15.3 (13.7, 17.0)
	2011	72	17.8	15.4 (13.6, 17.4)
	2012	89	21.4	15.6 (13.7, 17.9)
	2013	111	27.2	15.9 (14.0, 18.1)
	2014	97	23.5	16.2 (14.3, 18.3)
	2015	90	21.8	16.4 (14.2, 18.9)
	2016	90	21.5	16.7 (13.8, 20.1)
Scotland	2006	466	17.7	12.1 (11.1, 13.2)
	2007	408	15.2	11.1 (10.4, 11.9)
	2008	425	15.6	10.2 (9.6, 10.8)
	2009	367	13.2	9.4 (8.9, 10.0)
	2010	365	12.9	8.8 (8.3, 9.4)
	2011	356	12.4	8.5 (8.0, 9.0)
	2012	350	11.9	8.3 (7.8, 8.7)

Country/ region	Calendar year	Number of deaths	Crude rate (per 1000 person-years)	Age- and sex-standardised rate (per 1000 person-years, 95% CI)
	2013	350	11.7	8.1 (7.7, 8.6)
	2014	349	11.5	8.1 (7.5, 8.7)
	2015	383	12.4	8.0 (7.3, 8.8)
Spain (Catalonia)	2009	151	11.5	5.5 (4.6, 6.5)
	2010	122	8.9	5.3 (4.6, 6.1)
	2011	144	10.3	5.1 (4.5, 5.7)
	2012	103	7.1	4.9 (4.4, 5.5)
	2013	142	9.6	4.7 (4.3, 5.3)
	2014	84	5.6	4.6 (4.1, 5.2)
	2015	152	9.9	4.4 (3.8, 5.1)
	2016	133	8.5	4.3 (3.5, 5.1)
USA (KPNW)	2000	42	25.7	11.7 (8.4, 16.4)
	2001	35	23.3	11.2 (8.6, 14.6)
	2002	29	20.1	10.7 (8.4, 13.5)
	2003	23	16.8	10.2 (8.0, 13.0)
	2004	22	15.8	9.8 (7.6, 12.5)
	2005	30	21.2	9.4 (7.4, 11.9)
	2006	13	8.8	9.1 (7.3, 11.3)
	2007	20	13.5	8.8 (7.2, 10.8)
	2008	14	9.2	8.6 (7.0, 10.6)
	2009	20	13.0	8.4 (6.7, 10.6)
	2010	23	14.8	8.3 (6.6, 10.5)
	2011	18	11.1	8.2 (6.5, 10.3)
	2012	19	11.3	8.1 (6.5, 10.1)
	2013	23	13.7	8.1 (6.5, 10.0)
	2014	18	10.3	8.0 (6.3, 10.2)
	2015	20	11.0	8.0 (6.0, 10.6)
	2016	23	12.0	7.9 (5.6, 11.2)

Mortality rates are standardised to the age and sex distribution of the assembled population with type 1 diabetes within the six data sources, with equal weights for male and female individuals.

ESM Table 5 Annual estimated change in all-cause mortality rates in people with type 1 diabetes and annual estimated change in SMR

Country/ region	Annual estimated change in mortality rates (%, 95% CI)			Annual estimated change in SMR (%, 95% CI)		
	Total	Male	Female	Total	Male	Female
Australia	-2.1 (-2.8, -1.3)	-2.1 (-3.0, -1.1)	-2.1 (-3.3, -0.8)	-0.4 (-1.2, 0.4)	-0.2 (-1.2, 0.8)	-0.6 (-1.9, 0.7)
Denmark	-5.8 (-6.5, -5.1)	-6.0 (-6.8, -5.1)	-5.4 (-6.6, -4.2)	-2.4 (-3.2, -1.7)	-2.6 (-3.5, -1.7)	-2.0 (-3.3, -0.8)
Latvia	-2.2 (-3.6, -0.8)	-2.3 (-4.0, -0.6)	-2.1 (-4.5, 0.3)	1.1 (-0.4, 2.5)	1.0 (-0.7, 2.8)	1.0 (-1.4, 3.5)
Scotland	-4.8 (-5.8, -3.7)	-5.0 (-6.4, -3.6)	-4.5 (-6.1, -2.8)	-2.3 (-3.4, -1.2)	-2.3 (-3.7, -0.9)	-2.4 (-4.0, -0.7)
Spain (Catalonia)	-3.8 (-6.3, -1.2)	-4.0 (-7.1, -0.8)	-3.3 (-7.5, 1.1)	-6.3 (-8.8, -3.8)	-6.5 (-9.5, -3.3)	-6.1 (-10.2, -1.7)
USA (KPNW)	-3.4 (-5.3, -1.5)	-2.9 (-5.4, -0.2)	-4.1 (-6.8, -1.3)	0.0 (-1.9, 2.0)	0.8 (-1.9, 3.6)	-0.9 (-3.7, 2.0)

SMR, standardised mortality ratio.

ESM Fig. 1 Crude all-cause mortality rates in people with type 1 diabetes stratified by sex.

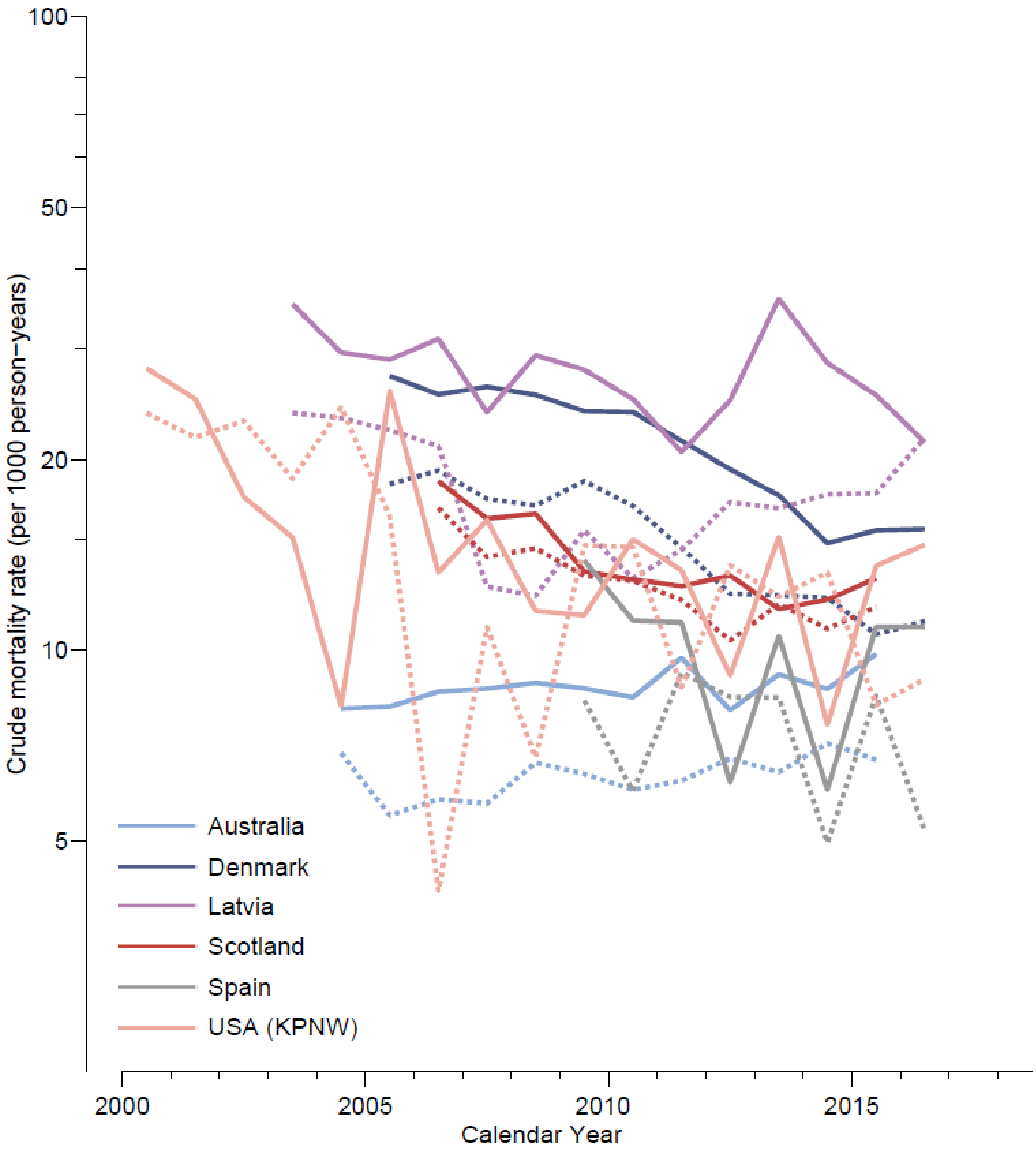

Male individuals in full lines, female individuals in broken lines. The *y*-axis is plotted on a natural logarithmic scale.

ESM Fig. 2 Age-standardised all-cause mortality rates in people with type 1 diabetes stratified by sex.

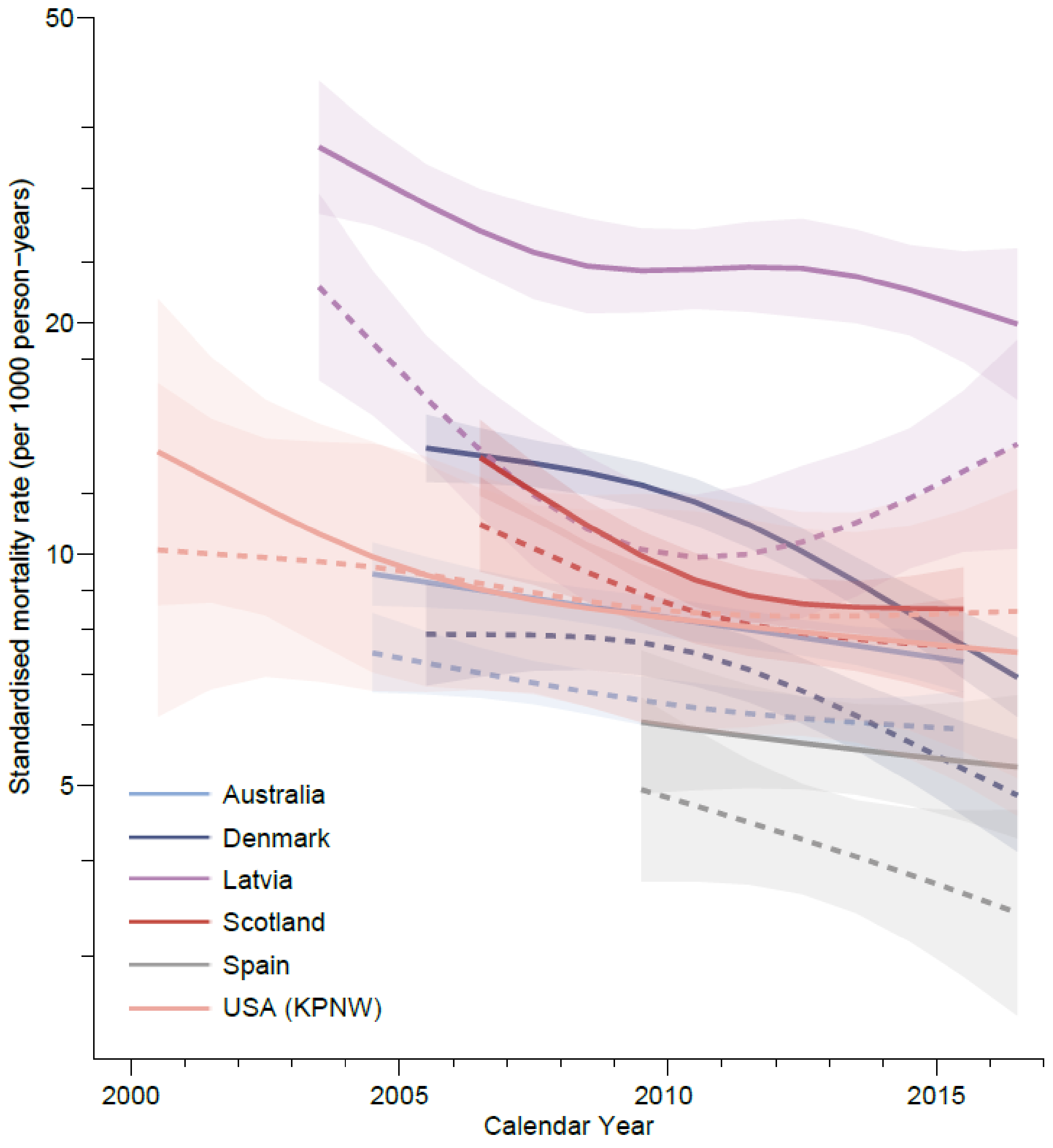

Male individuals in full lines, female individuals in broken lines. Standardisation is based on annual age-specific mortality rates from age-period-cohort models fitted separately for each data source and sex. Standard population was derived from the pooled study population with type 1 diabetes within the six data sources, with equal weights for male and female individuals. Shaded areas represent 95% CI around mortality trends. The *y*-axis is plotted on a natural logarithmic scale.

ESM Fig. 3 Estimated mortality rates in people with type 1 diabetes by age and period in Australia.

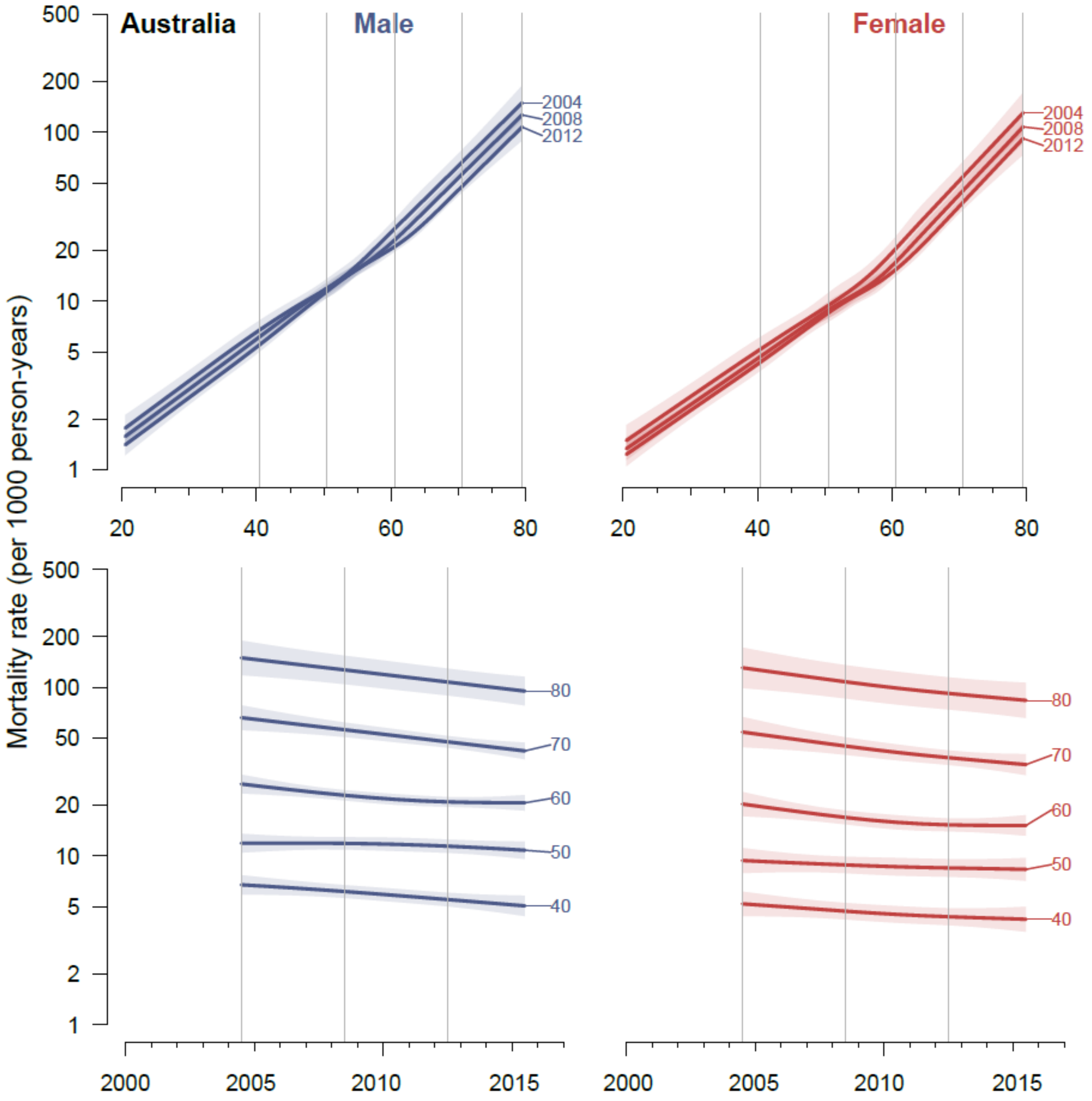

Male individuals in blue (left panels), female individuals in red (right panels). Estimates of mortality rates are from age–period–cohort models, fitted separately for male and female individuals. Upper panels show age-specific rates at different dates, as indicated by vertical lines in the lower panels. Lower panels show period-specific rates at different ages, as indicated by vertical lines in the upper panels. The *y*-axis is plotted on a natural logarithmic scale.

ESM Fig. 4 Estimated mortality rates in people with type 1 diabetes by age and period in Denmark.

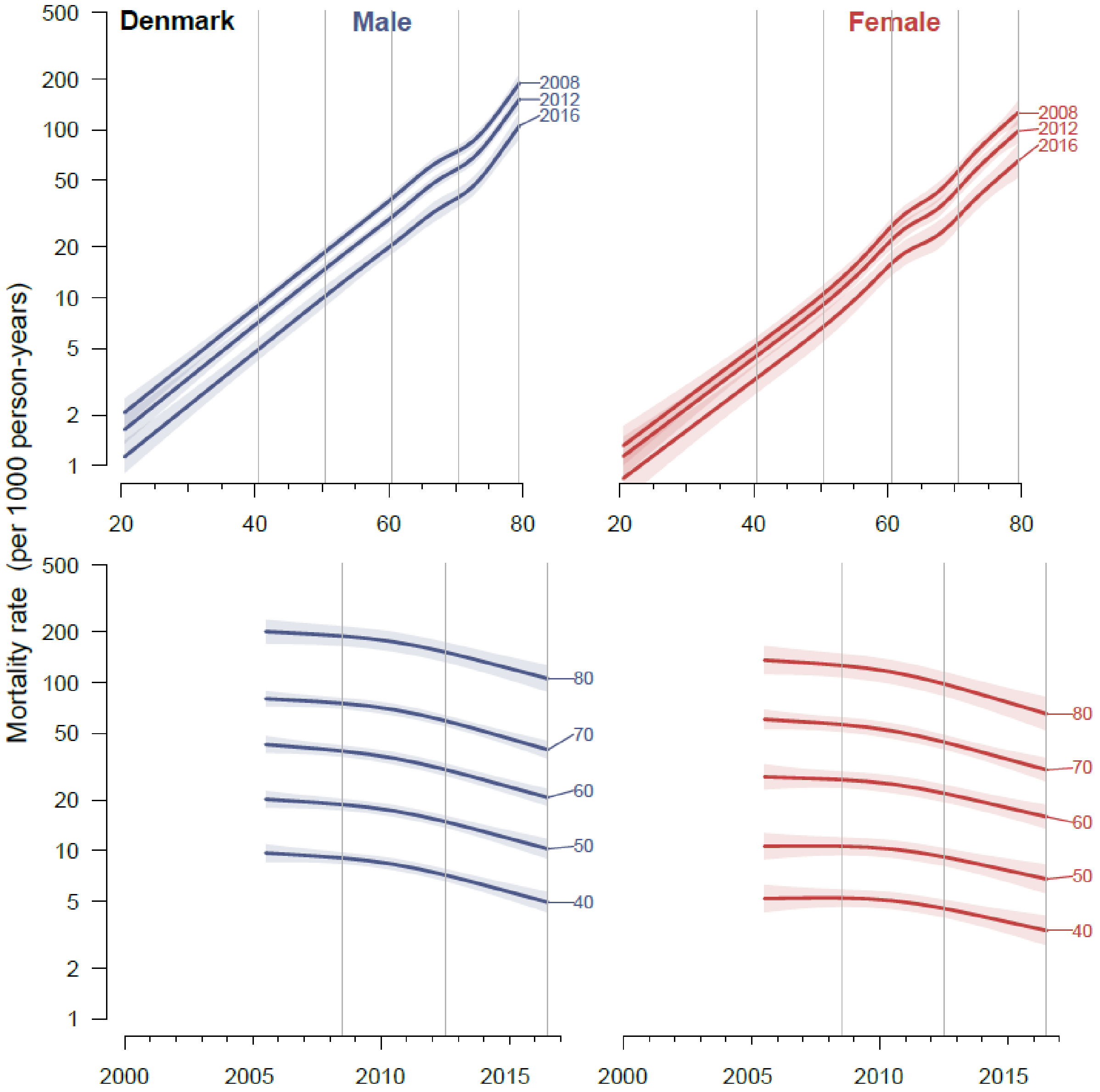

Male individuals in blue (left panels), female individuals in red (right panels). Estimates of mortality rates are from age–period–cohort models, fitted separately for male and female individuals. Upper panels show age-specific rates at different dates, as indicated by vertical lines in the lower panels. Lower panels show period-specific rates at different ages, as indicated by vertical lines in the upper panels. The *y*-axis is plotted on a natural logarithmic scale.

ESM Fig. 5 Estimated mortality rates in people with type 1 diabetes by age and period in Latvia.

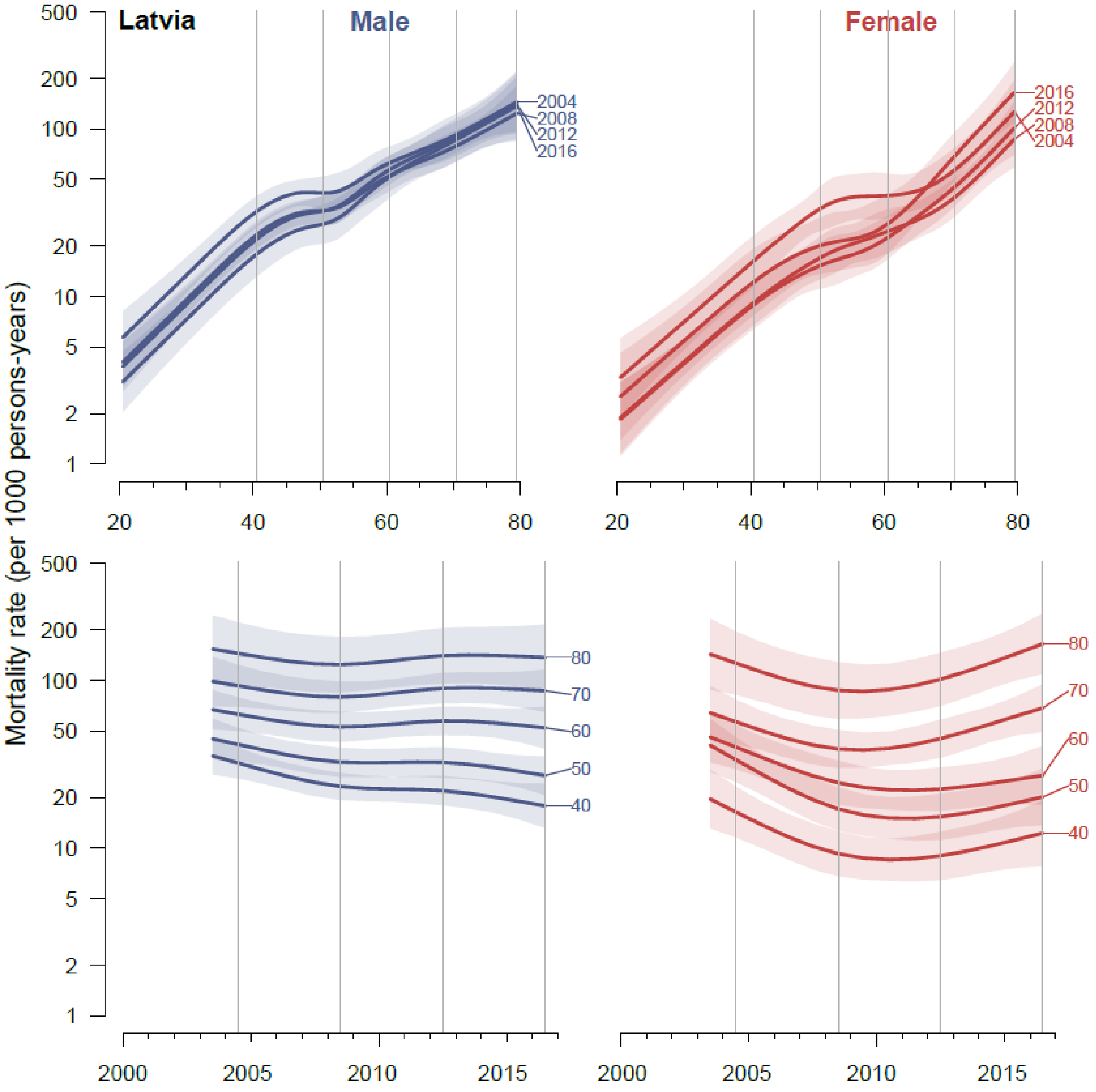

Male individuals in blue (left panels), female individuals in red (right panels). Estimates of mortality rates are from age–period–cohort models, fitted separately for male and female individuals. Upper panels show age-specific rates at different dates, as indicated by vertical lines in the lower panels. Lower panels show period-specific rates at different ages, as indicated by vertical lines in the upper panels. The *y*-axis is plotted on a natural logarithmic scale.

ESM Fig. 6 Estimated mortality rates in people with type 1 diabetes by age and period in Scotland.

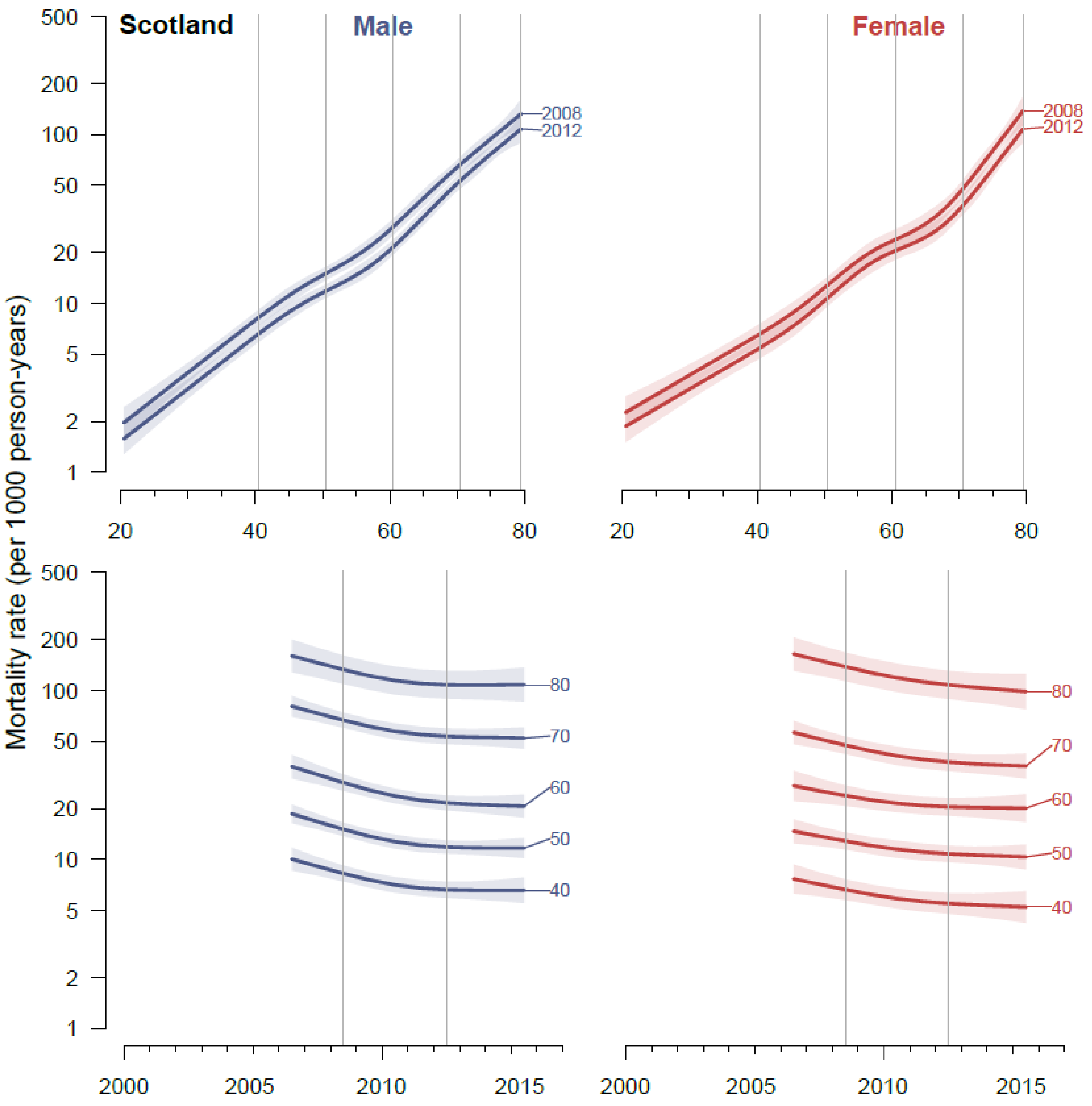

Male individuals in blue (left panels), female individuals in red (right panels). Estimates of mortality rates are from age–period–cohort models, fitted separately for male and female individuals. Upper panels show age-specific rates at different dates, as indicated by vertical lines in the lower panels. Lower panels show period-specific rates at different ages, as indicated by vertical lines in the upper panels. The *y*-axis is plotted on a natural logarithmic scale.

ESM Fig. 7 Estimated mortality rates in people with type 1 diabetes by age and period in Spain.

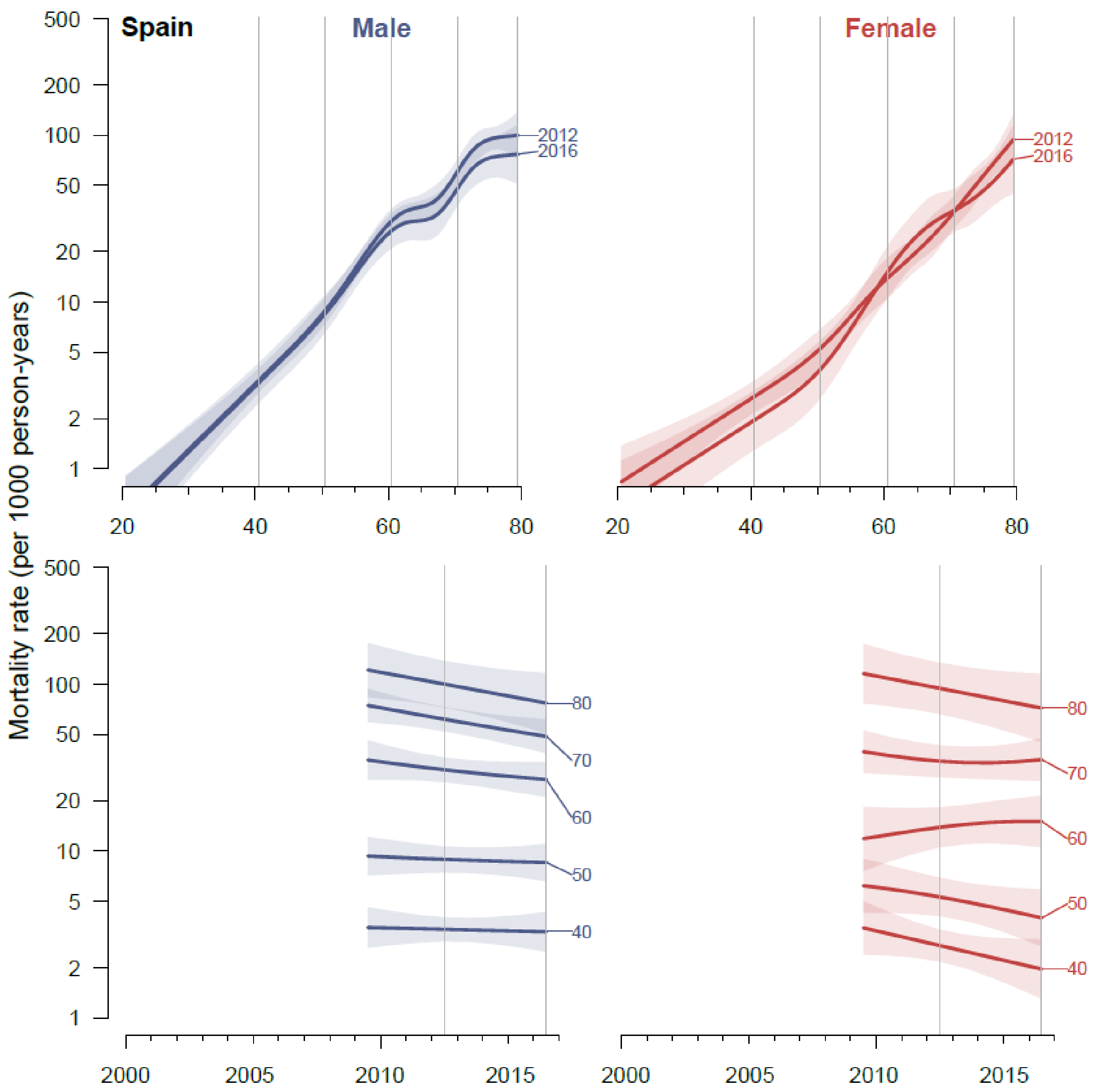

Male individuals in blue (left panels), female individuals in red (right panels). Estimates of mortality rates are from age–period–cohort models, fitted separately for male and female individuals. Upper panels show age-specific rates at different dates, as indicated by vertical lines in the lower panels. Lower panels show period-specific rates at different ages, as indicated by vertical lines in the upper panels. The *y*-axis is plotted on a natural logarithmic scale.

ESM Fig. 8 Estimated mortality rates in people with type 1 diabetes by age and period in the USA (KPNW).

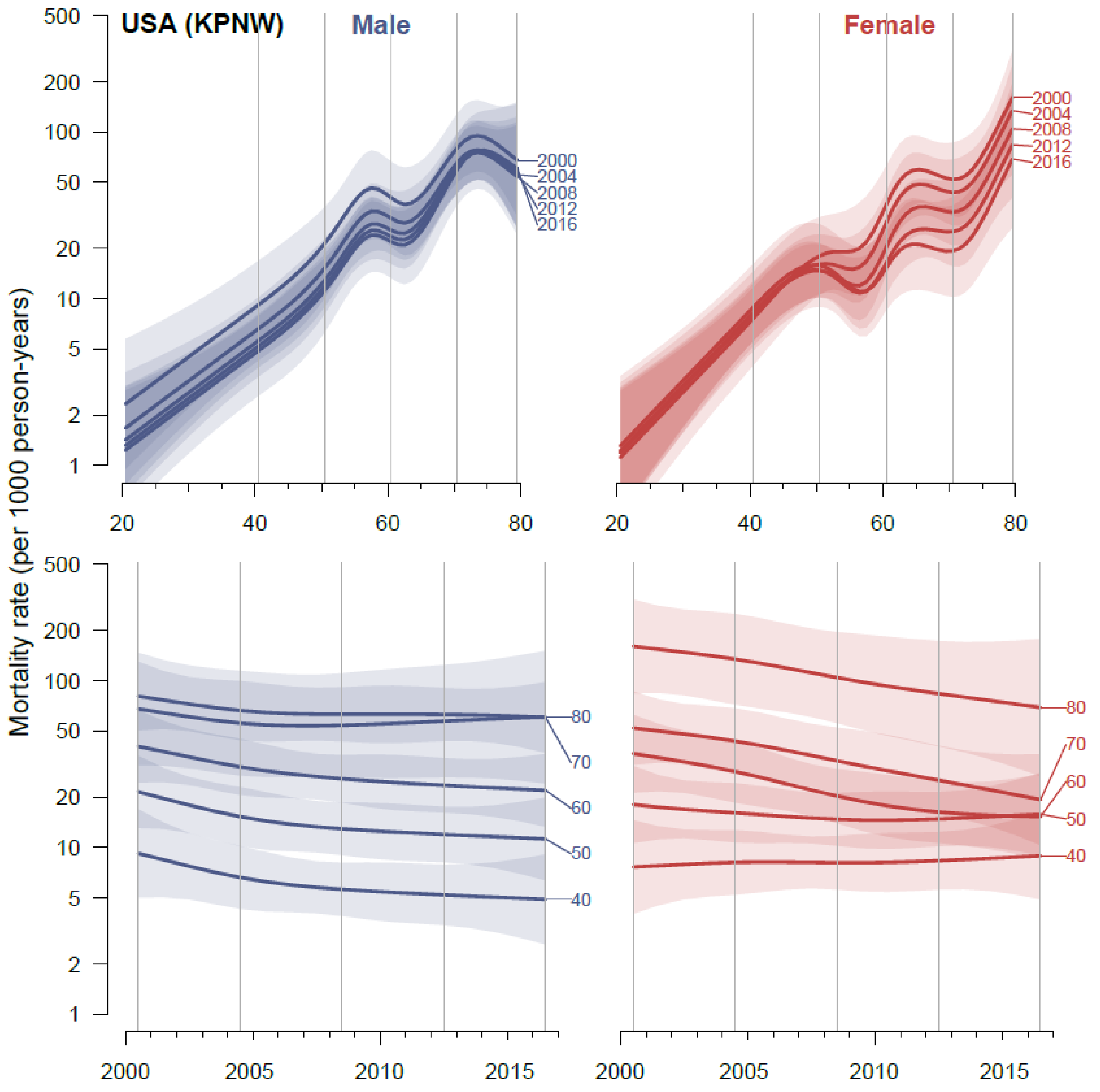

Male individuals in blue (left panels), female individuals in red (right panels). Estimates of mortality rates are from age–period–cohort models, fitted separately for male and female individuals. Upper panels show age-specific rates at different dates, as indicated by vertical lines in the lower panels. Lower panels show period-specific rates at different ages, as indicated by vertical lines in the upper panels. The *y*-axis is plotted on a natural logarithmic scale.

ESM Fig. 9 Age by time interaction of mortality rates as estimated annual change in mortality by age.

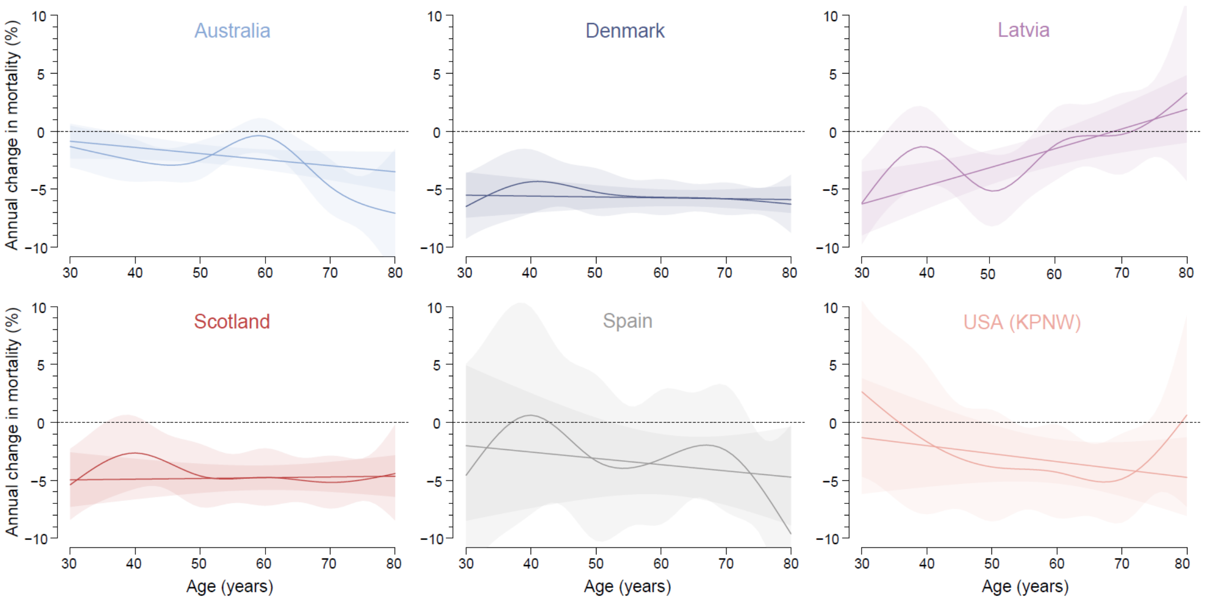

Estimated annual change in mortality was computed from a model with calendar time trend varying by age, either by smooth splines or linearly; both plotted in each panel. The curves were derived using a spline for age (a varying coefficients model), while the straight lines using the product of age and calendar time.

ESM Fig. 10 SMR in people with type 1 diabetes compared with those without diabetes stratified by sex.

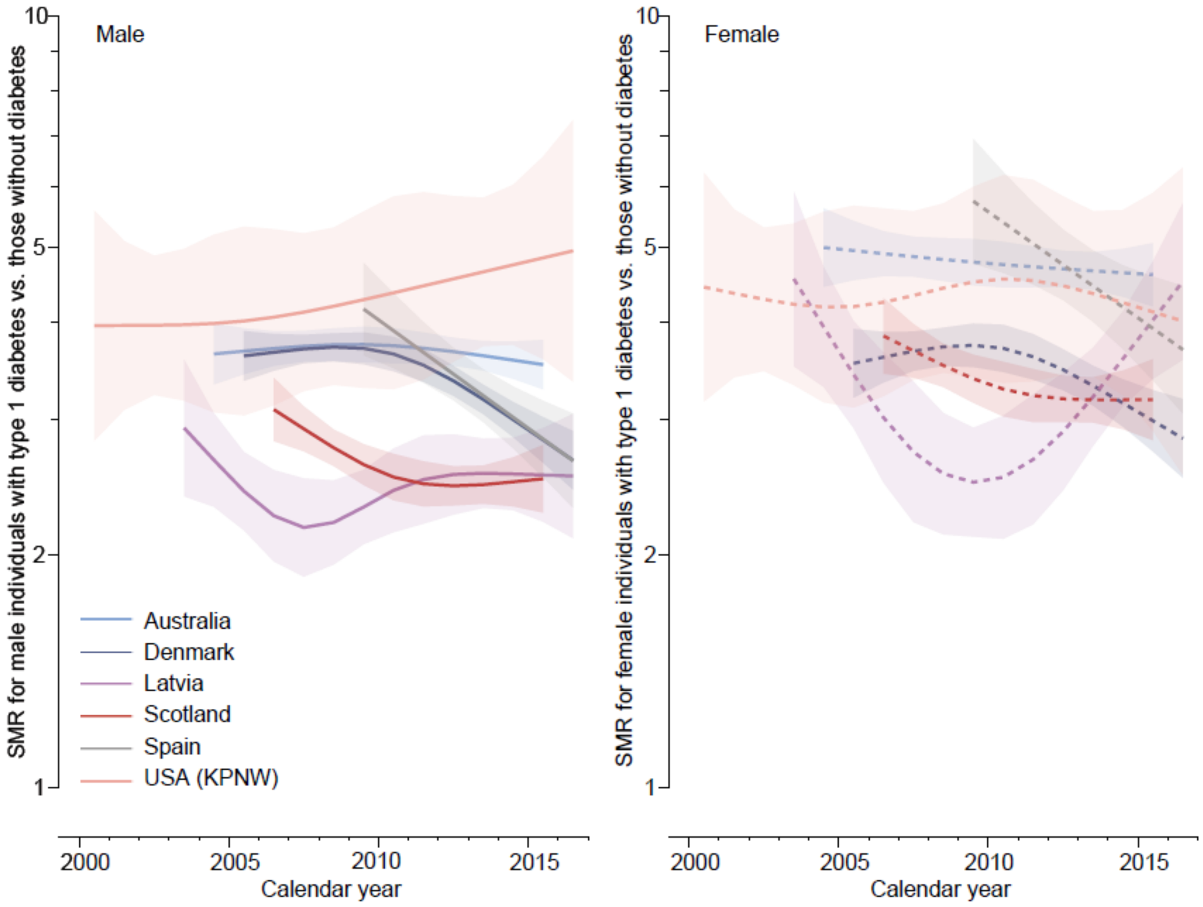

Male individuals in full lines, female individuals in broken lines. Smoothing is based on a model with SMR constant over age. SMR, standardised mortality ratio. The *y*-axis is plotted on a natural logarithmic scale.

ESM Fig. 11 Estimated SMR in people with type 1 diabetes compared with those without diabetes by age and period in Australia.

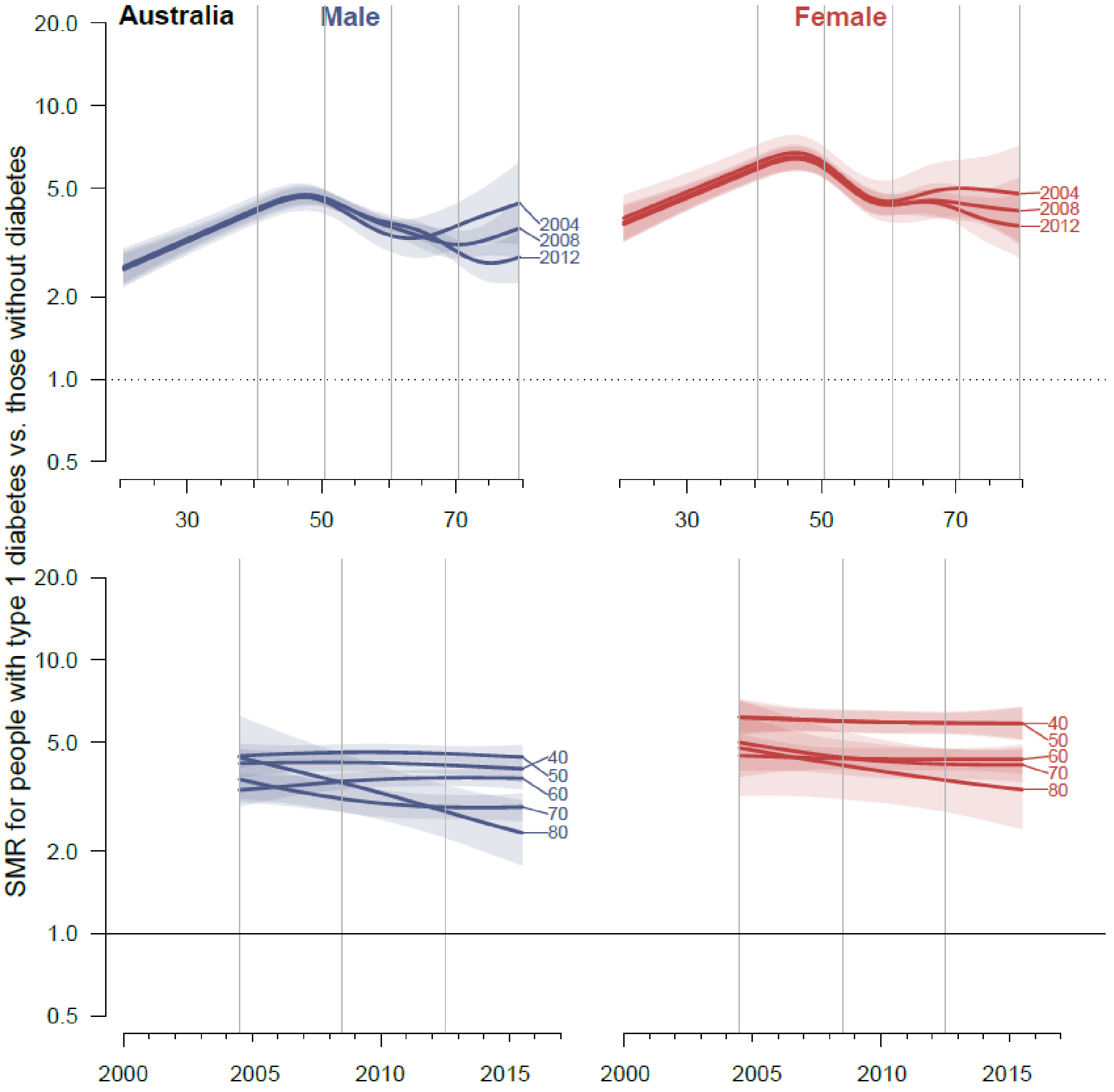

Male individuals in blue (left panels), female individuals in red (right panels). Estimates of the SMRs are from age–period–cohort models, fitted separately for male and female individuals. Upper panels show age-specific SMRs at different dates, as indicated by vertical lines in the lower panels. Lower panels show period-specific SMRs at different ages, as indicated by vertical lines in the upper panels. SMR, standardised mortality ratio. The *y*-axis is plotted on a natural logarithmic scale.

ESM Fig. 12 Estimated SMR in people with type 1 diabetes compared with those without diabetes by age and period in Denmark.

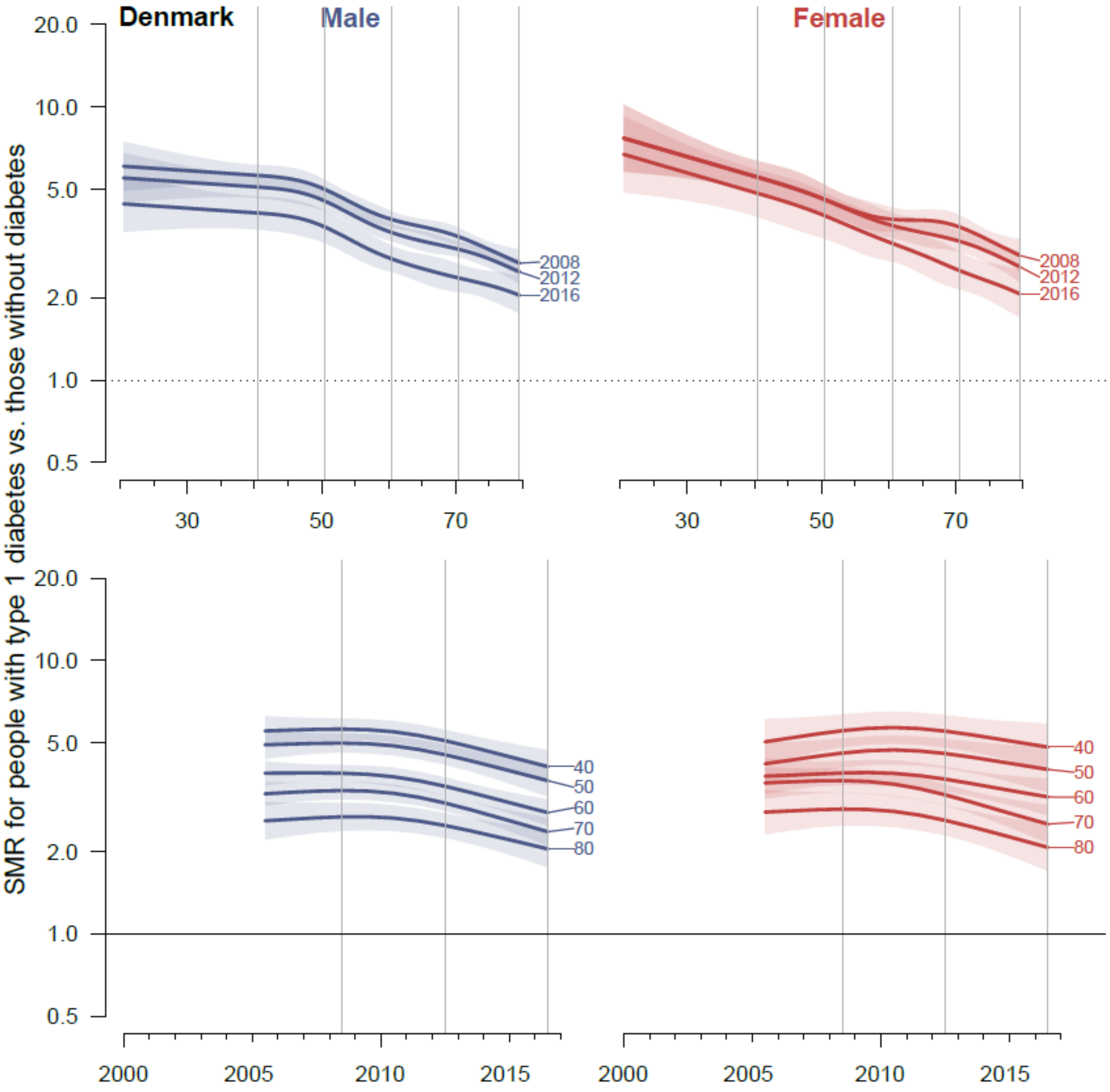

Male individuals in blue (left panels), female individuals in red (right panels). Estimates of the SMRs are from age–period–cohort models, fitted separately for male and female individuals. Upper panels show age-specific SMRs at different dates, as indicated by vertical lines in the lower panels. Lower panels show period-specific SMRs at different ages, as indicated by vertical lines in the upper panels. SMR, standardised mortality ratio. The *y*-axis is plotted on a natural logarithmic scale.

ESM Fig. 13 Estimated SMR in people with type 1 diabetes compared with those without diabetes by age and period in Latvia.

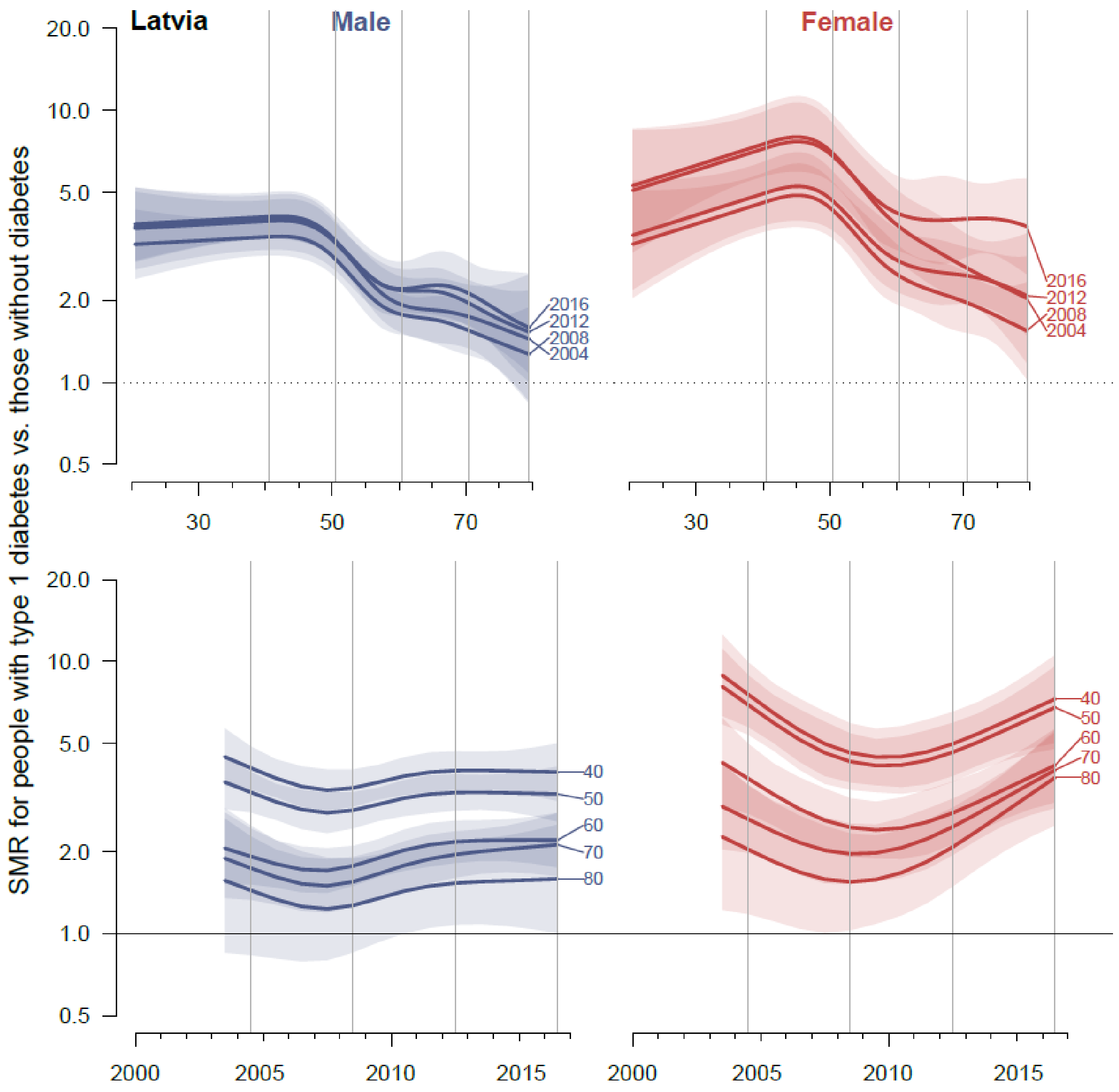

Male individuals in blue (left panels), female individuals in red (right panels). Estimates of the SMRs are from age–period–cohort models, fitted separately for male and female individuals. Upper panels show age-specific SMRs at different dates, as indicated by vertical lines in the lower panels. Lower panels show period-specific SMRs at different ages, as indicated by vertical lines in the upper panels. SMR, standardised mortality ratio. The *y*-axis is plotted on a natural logarithmic scale.

ESM Fig. 14 Estimated SMR in people with type 1 diabetes compared with those without diabetes by age and period in Scotland.

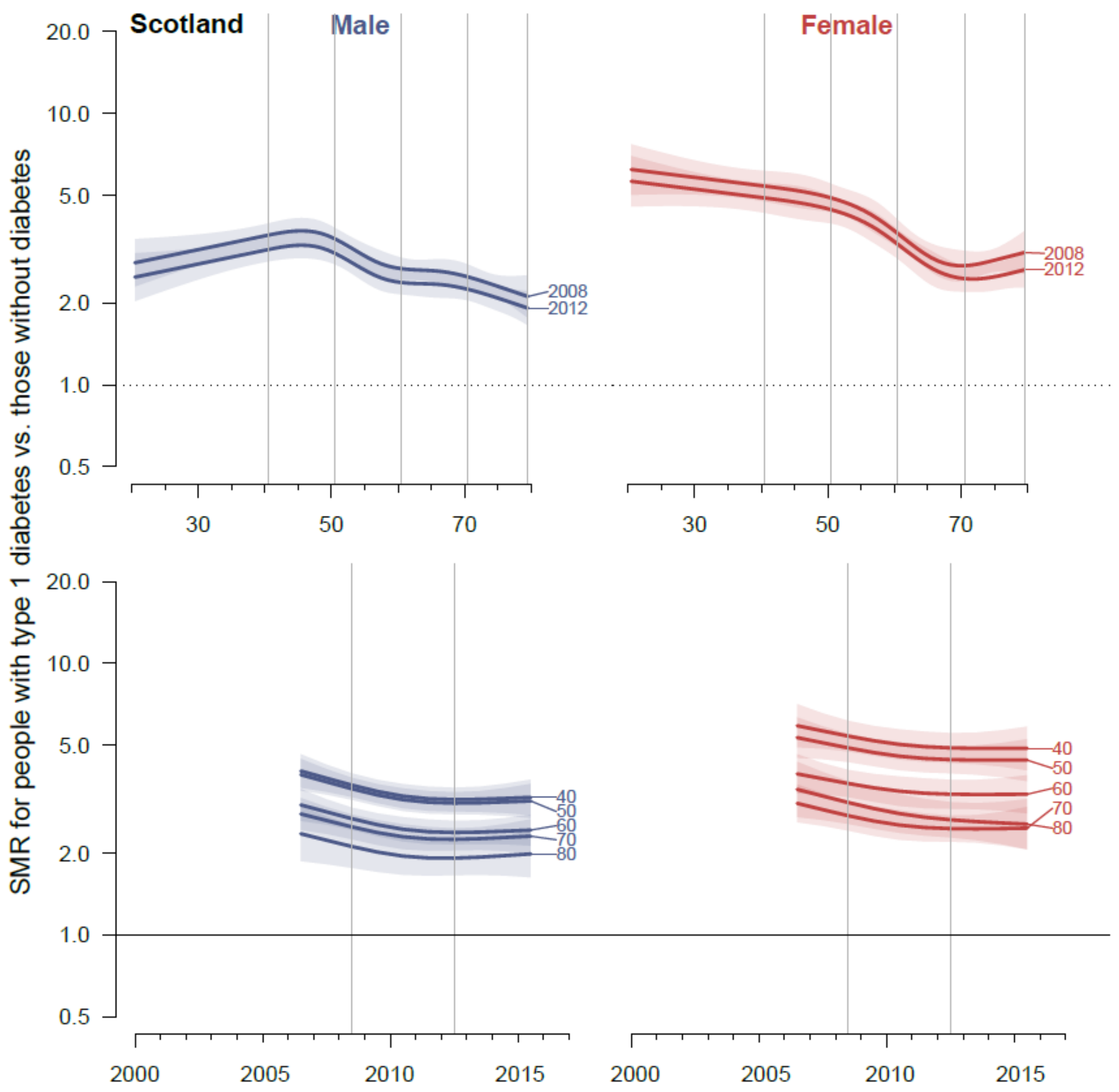

Male individuals in blue (left panels), female individuals in red (right panels). Estimates of the SMRs are from age–period–cohort models, fitted separately for male and female individuals. Upper panels show age-specific SMRs at different dates, as indicated by vertical lines in the lower panels. Lower panels show period-specific SMRs at different ages, as indicated by vertical lines in the upper panels. SMR, standardised mortality ratio. The *y*-axis is plotted on a natural logarithmic scale.

ESM Fig. 15 Estimated SMR in people with type 1 diabetes compared with those without diabetes by age and period in Spain.

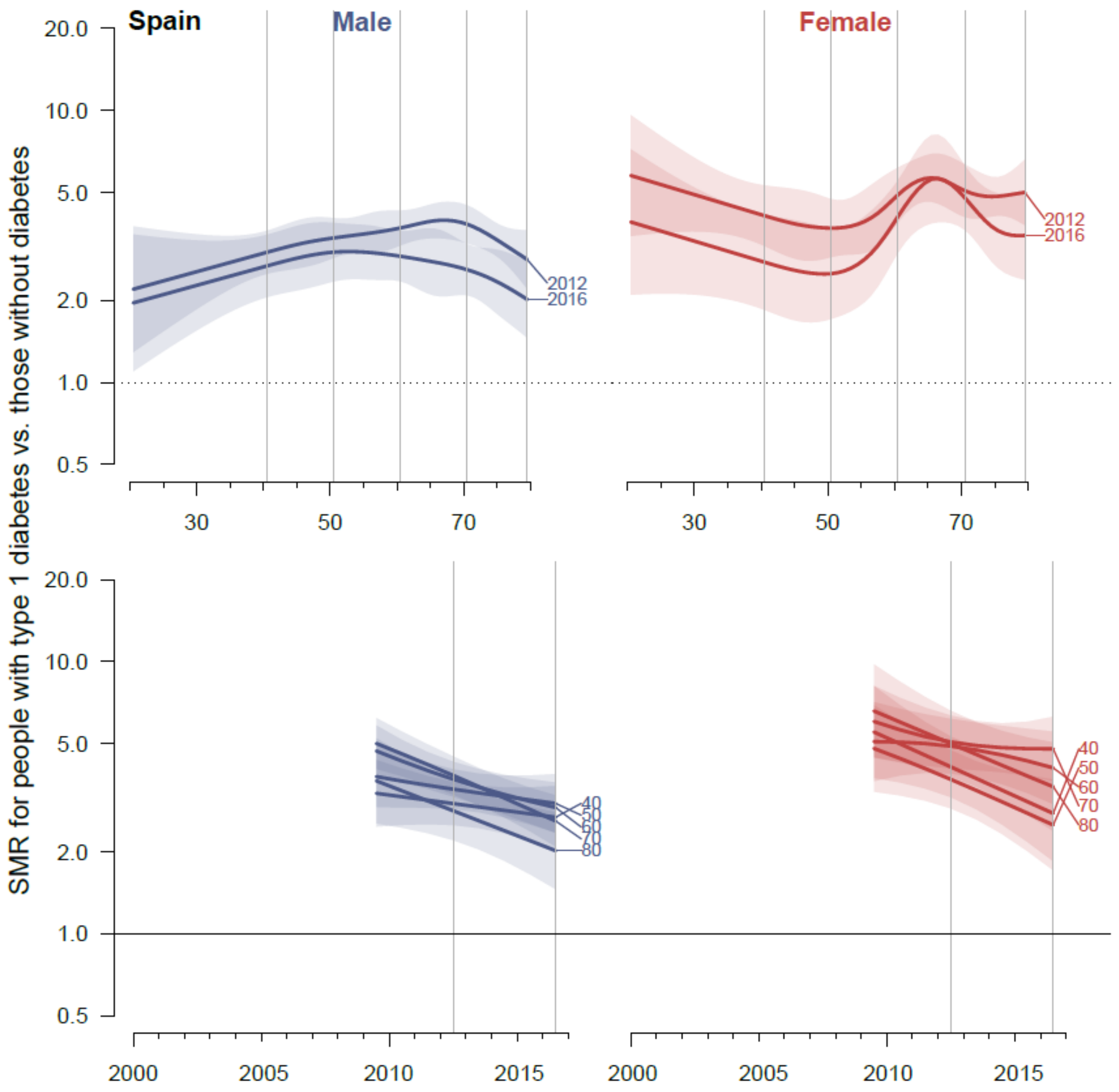

Male individuals in blue (left panels), female individuals in red (right panels). Estimates of the SMRs are from age–period–cohort models, fitted separately for male and female individuals. Upper panels show age-specific SMRs at different dates, as indicated by vertical lines in the lower panels. Lower panels show period-specific SMRs at different ages, as indicated by vertical lines in the upper panels. SMR, standardised mortality ratio. The *y*-axis is plotted on a natural logarithmic scale.

ESM Fig. 16 Estimated SMR in people with type 1 diabetes compared with those without diabetes by age and period in the USA (KPNW).

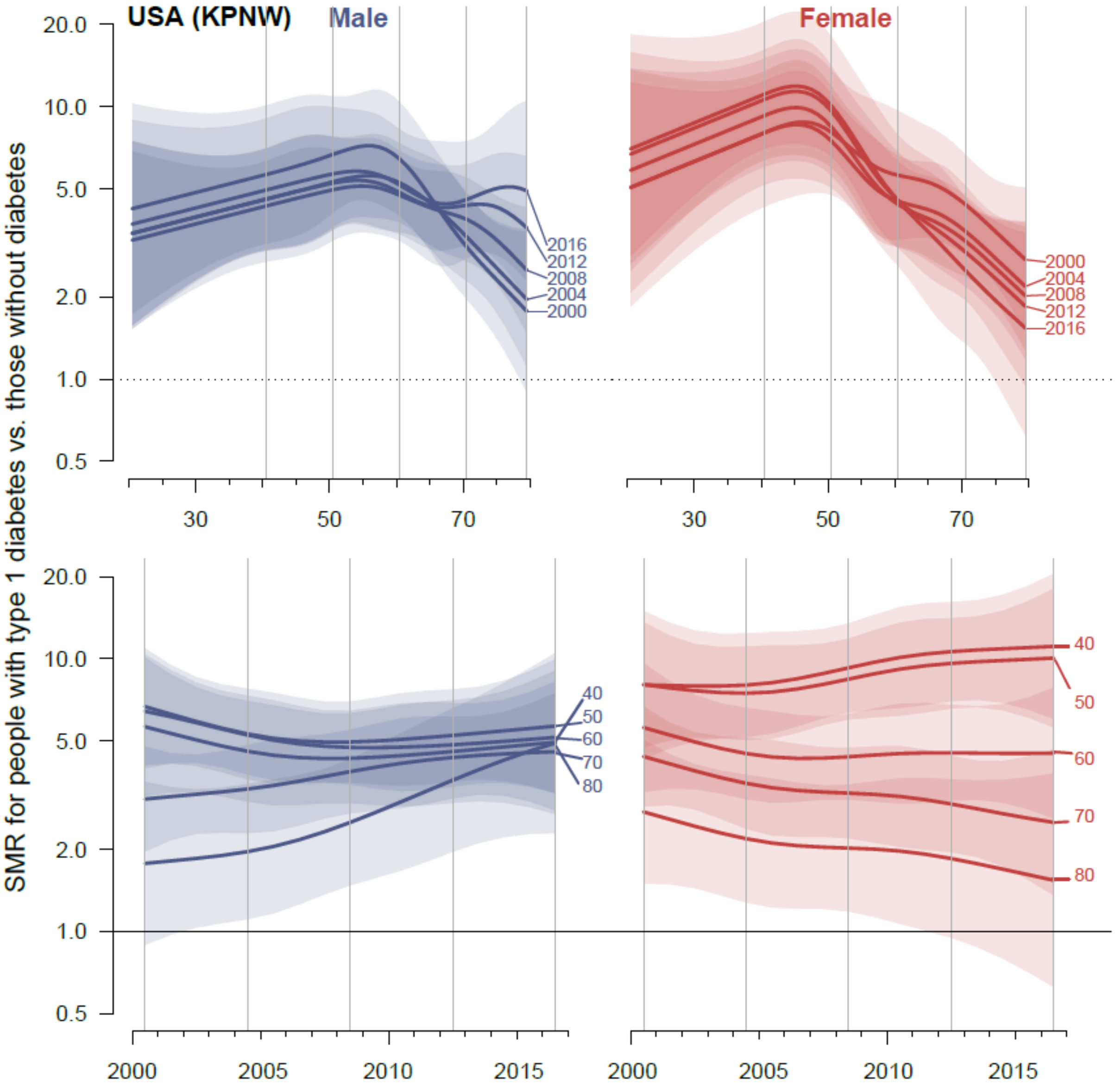

Male individuals in blue (left panels), female individuals in red (right panels). Estimates of the SMRs are from age–period–cohort models, fitted separately for male and female individuals. Upper panels show age-specific SMRs at different dates, as indicated by vertical lines in the lower panels. Lower panels show period-specific SMRs at different ages, as indicated by vertical lines in the upper panels. SMR, standardised mortality ratio. The *y*-axis is plotted on a natural logarithmic scale.

References

1. Sacre JW, Harding JL, Shaw JE, Magliano DJ (2021) Declining mortality in older people with type 2 diabetes masks rising excess risks at younger ages: a population-based study of all-cause and cause-specific mortality over 13 years. Int J Epidemiol 50(4): 1362-1372. https://doi.org/10.1093/ije/dyaa270

2. Carstensen B, Ronn PF, Jorgensen ME (2020) Prevalence, incidence and mortality of type 1 and type 2 diabetes in Denmark 1996-2016. BMJ Open Diabetes Res Care 8(1): e001071. https://doi.org/10.1136/bmjdrc-2019-001071

3. O'Reilly JE, Blackbourn LAK, Caparrotta TM et al (2020) Time trends in deaths before age 50 years in people with type 1 diabetes: a nationwide analysis from Scotland 2004-2017. Diabetologia 63(8): 1626-1636. https://doi.org/10.1007/s00125-020-05173-w

4. Schroeder EB, Donahoo WT, Goodrich GK, Raebel MA (2018) Validation of an algorithm for identifying type 1 diabetes in adults based on electronic health record data. Pharmacoepidemiol Drug Saf 27(10): 1053-1059. https://doi.org/10.1002/pds.4377
